# Heritability Estimation using a Regularized Regression Approach (HERRA): Applicable to continuous, dichotomous or age-at-onset outcome

**DOI:** 10.1371/journal.pone.0181269

**Published:** 2017-08-16

**Authors:** Malka Gorfine, Sonja I. Berndt, Jenny Chang-Claude, Michael Hoffmeister, Loic Le Marchand, John Potter, Martha L. Slattery, Nir Keret, Ulrike Peters, Li Hsu

**Affiliations:** 1 Department of Statistics and Operations Research, Tel Aviv University, Tel Aviv, Israel; 2 Division of Cancer Epidemiology and Genetics, National Cancer Institute, National Institutes of Health, Bethesda, Maryland, United States of America; 3 Division of Cancer Epidemiology, German Cancer Research Center, Heidelberg, Germany; 4 Division of Clinical Epidemiology and Aging Research, German Cancer Research Center, Heidelberg, Germany; 5 Epidemiology Program, University of Hawaii Cancer Center, Honolulu, Hawaii, United States of America; 6 Public Health Sciences Division, Fred Hutchinson Cancer Research Center, Seattle, Washington, United States of America; 7 Department of Internal Medicine, University of Utah Health Sciences Center, Salt Lake City, Utah, United States of America; New Jersey Institute of Technology, UNITED STATES

## Abstract

The popular Genome-wide Complex Trait Analysis (GCTA) software uses the random-effects models for estimating the narrow-sense heritability based on GWAS data of unrelated individuals without knowing and identifying the causal loci. Many methods have since extended this approach to various situations. However, since the proportion of causal loci among the variants is typically very small and GCTA uses all variants to calculate the similarities among individuals, the estimation of heritability may be unstable, resulting in a large variance of the estimates. Moreover, if the causal SNPs are not genotyped, GCTA sometimes greatly underestimates the true heritability. We present a novel narrow-sense heritability estimator, named HERRA, using well-developed ultra-high dimensional machine-learning methods, applicable to continuous or dichotomous outcomes, as other existing methods. Additionally, HERRA is applicable to time-to-event or age-at-onset outcome, which, to our knowledge, no existing method can handle. Compared to GCTA and LDAK for continuous and binary outcomes, HERRA often has a smaller variance, and when causal SNPs are not genotyped, HERRA has a much smaller empirical bias. We applied GCTA, LDAK and HERRA to a large colorectal cancer dataset using dichotomous outcome (4,312 cases, 4,356 controls, genotyped using Illumina 300K), the respective heritability estimates of GCTA, LDAK and HERRA are 0.068 (SE = 0.017), 0.072 (SE = 0.021) and 0.110 (SE = 5.19 x 10^−3^). HERRA yields over 50% increase in heritability estimate compared to GCTA or LDAK.

## Introduction

Heritability is a concept that summarizes the proportion of phenotypic variance that is due to genetic factors, with broad-sense heritability referring to genetic variation that may include effects due to additive genetic variation as well as dominance and epistasis, and narrow-sense heritability, *h*^2^, referring to additive genetic variation only [1]. Breakthroughs in high throughput technologies have enables researchers to conduct large-scale genome-wide associate studies for many complex diseases. A question of key interest is to estimate the (narrow-sense) heritability from the genome-wide genotyped data and have an overall assessment of the extent of genetic components associated with complex traits, providing guidance for future discoveries of genetic loci.

Random-effects models were used for heritability esitmation by animal breeders decades ago, and have recently been introduced into human genetics by Yang et al. [2] to estimate heritability based on genome-wide association studies (GWASs) of apparently unrelated individuals. This approach, known as genomic restricted maximum likelihood (GREML), is applied by the Genome-wide Complex Trait Analysis (GCTA) software [3]. Since additional fixed effects (e.g. sex) can also be included in the model, it is often referred to as a mixed-effects model approach. The key advantage of their approach is that they allow estimation of the overall heritability of traits without explicitly identifying causal loci. In this approach [2], each subject’s trait is controlled by genetic random effects that are correlated across subjects by virtue of sharing some of the genetic variants affecting the trait, and by an environmental random effect that is uncorrelated among subjects. Since the identity of the causal SNPs is unknown, applying the standard maximum likelihood method for estimating the model parameters is impossible. Instead, Yang et al. [2] heuristically approximated the genetic correlation between each pair of subjects across the causal SNPs by the observed correlation matrix of all genotyped SNPs. To account for linkage disequilibrium (LD) between genotyped SNPs and causal SNPs they heuristically corrected the observed correlation matrix using simulations.

Zaitlen et al. [4] extended the random-effects approach of Yang et al. [2] and provided an identical-by-descent-based heritability estimator with closely and distantly related pairs of individuals. Golan and Rosset [5] and Speed et al. [6] indicated that the efficiency of Yang et al.’s [2] method seriously deteriorates as the proportion of causal SNPs decreases. It was shown [5] that since most of the genotyped SNPs are not causative, the very large number of SNPs used for estimating the genetic correlations masks the correlation on the set of causal SNPs, which can lead to inefficient heritability estimation. Instead, Golan and Rosset [5] proposed treating the identity of causal SNPs as missing data, and obtained the maximum likelihood estimator based on the computationally-intensive Markov Chain Monte Carlo method. However, this approach is not tractable computationally for situations consisting of ∼300K or more genotyped SNPs, as considered here. Moser et al. [7] also extended Yang et al.’s approach and used a Baysian mixture model of four normal distributions of the SNPs’ random effects instead of just one distribution.

Speed et al. [6] raised another concern in which uneven LD between SNPs can generate a large bias in the heritability estimator based on the mixed effects model approach. Causal variants tend to be overestimated in regions of strong LD and underestimated in regions of low LD. In practice, if some of the causal variants are being tagged by multiple genotyped SNPs more than others, it distorts their contributions to the heritability estimator. Hence, they proposed to overcome the problem by replacing the observed correlation matrix by a weighted matrix consisting of scaling SNP genotypes according to local LD patterns. The weights are identified using a linear programming procedure. This approach can be applied by the LDAK software.

All the available methods for heritability estimation based on GWAS data of unrelated individuals use continuous or dichotomous outcomes. A cohort dataset of a certain disease usually consists of age-at-onset (or age at diagnosis) for diseased individuals, and current age or age at death for disease-free individuals, known as survival outcome. The highly cited paper [8] studied heritability of prostate cancer and reported heritability estimates of 0.42 (95% confidence limits 0.29–0.50) based on combined cohorts of 44,788 twin pairs from the Nordic twin registries, suggesting a considerable genetic contribution to the development of prostate cancer. This estimate is based on a polygenic liability-threshold model, quantified the heritability on the liability scale, while ignoring the observed ages at onset and instead, classifying subjects as cancer or cancer free (dichotomous outcome). Cancer-free individuals include subjects who died without cancer, and those who were still alive but had not had cancer by the end of the follow-up period. Since about 70% of the individuals were still alive and cancer-free at the end of follow-up (known as right-censored observations), and were treated as cancer free for the rest of their life, the heritability estimates of the targeted population in this study could be severely biased. Indeed, Scheicke et al. [9] showed that heritability estimator based on a liability-threshold model of cohort twins data, yields a biased estimator, where the bias may go in either directions, and strongly depends on the censoring rate in a non-linear manner. Holst et al. [10] estimated prostate cancer heritability based on 15,509 male twins of Danish cohort twins, and showed that the liability-based heritability estimate which wrongly ignores right-censoring equals 0.73 (0.64–0.81), while using survival-analysis methods that correctly considered censored observations yields heritability estimate of 0.63 (0.49–0.77).

In this work, we provide a new heritability estimation approach using ultra-high dimensional machine-learning methods. The approach can be applied to not only continuous or dichotomous types of outcome, but also to time-to-event outcome, where right censoring is properly accommodated. We show through extensive simulation study that our proposed estimators have little bias for all outcomes, continuous, dichotomous and time-to-event. For continuous and dichotomous outcomes, we show that our proposed estimators are more efficient than that of GCTA and LDAK; and when causal SNPs are not tagged well, GCTA and LDAK yield under-estimated heritability, while the proposed heritability estimator has only a very small empirical bias. In practice, an estimator of a parameter is constructed as a function of the sample of size *N*. An important question is what the limit of a sequence of estimates (indexed by *N*) would be as the sample size *N* increased to infinity; a desirable property of an estimator is that this sequence of estimates converges to the true parameter value. Such an estimator is called a consistent estimator. We show that our heritability estimators are consistent.

Applying GCTA, LDAK and HERRA in case-control colorectal cancer data using binary outcome (4,312 cases and 4,356 controls genotyped using Illumina 300K), the respective heritability estimates of GCTA, LDAK and HERRA are 0.068 (SE = 0.017), 0.072 (SE = 0.021) and 0.110 (SE = 5.19 x 10^−3^). HERRA yields over a 50% increase in heritability estimate compared to GCTA or LDAK with substantially smaller standard error, and is closer to the heritability estimates obtained from twins and family data, which range from 0.12 to 0.35 [11]. This is probably due to the fact that GWAS SNPs are tagging SNPs and based on our simulation results presented in this paper, the GCTA or LDAK estimates are biased downward, whereas our proposed estimates generally have a very small empirical bias. A simple R code for HERRA used for analyzing the GECCO data is provided at http://www.tau.ac.il/~gorfinem/, and a friendly R package for applying HERRA will be released soon.

## Results

### Case-control colorectal cancer data

We applied the proposed method, HERRA, and the GCTA [3] to a large genome-wide association consortium, Genetics and Epidemiology of Colorectal Cancer Consoritum (GECCO) (Peters et al. 2012). Colorectal cancer (CRC) is one of the most commonly diagnosed cancers, and it remains the second leading cause of cancer death. It has a sizable genetic component and well-established lifestyle and environmental risk factors. In this consoritum, various genotyping platforms (Illumina 300K, Illumina 550K, combined Illumina 300K&240K, Illumina 610K, or Illumina 730K chips) have been used over time. For this illustration we focused on the largest subset of samples that were genotyped using the common platform, Illumina 300K.

Briefly, samples were excluded based on call rate, heterozygosity, unexpected duplicates, sex discrepancy, and unexpectedly high identity-by-descent or unexpected genotype concordance (> 65%) with another individual. All analyses were restricted to samples clustering with the Utah residents with Northern and Western European ancestry in HapMap II based on principal component analysis. SNPs were excluded if they were triallelic, not assigned an rs number, or were reported or observed as not performing consistently across platforms. Additionally, genotyped SNPs were excluded based on call rate (< 98%) and lack of Hardy Weinberg Equilibrium in controls (HWE, p < 1 × 10^−4^). In summary, there were a total of 4,312 cases and 4,356 controls, and 248,977 SNPs with minor allele frequency (MAF) > 0.01. Cases and controls were frequency-matched by age and sex. The mean age at onset was approximately 65 years old and there were slightly fewer women than men except for the WHI study where all were women (Table 1). The details of the studies are provided in S4 and S5 Text.

**Table 1:** Studies within GECCO used for heritability estimation.

Study	Case	Control	Female		Age (yrs)	
	N = 4312	N = 4356	No.	%	Mean	Range
Colo2&3	87	125	95	44.8	65.2	38–86
DACHS1	1710	1708	1395	40.8	68.6	33–98
DALS2	410	464	414	47.4	65.4	30–79
MEC	328	346	313	46.4	63.0	45–76
PLCO2	486	415	383	42.5	63.6	55–75
VITAL	285	288	273	47.6	66.5	50–76
WHI2	1006	1010	2016	100	65.8	50–79

HERRA estimator consists of four main steps, described in details in Methods, which forms the basis of our methodology. We first conducted iteratively thresholded ridge regression screener [12] (ITRRS) using a linear model to the CRC data, to reduce the dimensionality of the SNPs below the sample size *N* = 8,668, but still keeping the number of SNPs at this step to be of a relatively large scale. In this step, we performed the iteratively thresholded ridge regression for 6 iterations, keeping the top 50% SNPs after each iteration, based on the absolute value of the regression estimates. The ITRRS was performed for each choromosome separately and the selected SNPs were combined. This completes Step 1 of our procedure. Since we started with 248,977 SNPs, we assume sparsity such that the median of the ridge-regression coefficients within each chromosome equals zero. Therefore, no asymptotic bias is introduced by this dimensionality reduction step [13].

Using the selected SNPs by Step 1, we applied Step 2: the sample was randomly split into two equals subsets, and we applied lasso (with 10-fold cross validation and the minimum mean cross-validated error) for the first subset using a linear model with the SNPs of Step 1, yielded a parsimonious model with a small set of selected SNPs. By the ordinary least-squares method to the second subset, using only the selected SNPs, we obtained unbiased estimates of the regression coefficients and the variance of the error term, $\sigma_{\epsilon }^{2}$. In Step 3 we repeated Step 2 while switching the role of the first and second subset. The final estimate of $\sigma_{\epsilon }^{2}$, ${\hat{\sigma }}_{\epsilon }^{2}$, was obtained by the mean of the above two estimates obtained in Steps 2 and 3 which completes Step 4. Finally, HERRA estimate of the narrow-sense observed-scale heritability, as shown in Methods section yielded ${\hat{h}}_{o}^{2}=1-{\hat{\sigma }}_{\epsilon }^{2}/{\hat{\sigma }}_{D}^{2}=0.244$, where ${\hat{\sigma }}_{D}^{2}$ is an empirical estimator of the total variance of the binary outcome—the presence or absence of CRC. By the Robertson transformation [14] (see Methods), and the ascertainment correction of [15], which is appropriate under low heritability values [16], the narrow-sense heritability in liability scale was ${\hat{h}}_{l}^{2}={\hat{h}}_{o}^{2}K^{2}\left(1-K^{2}\right)/\left\{P\left(1-P\right)z^{2}\right\}=0.110$, where *P* is the disease-prevalence in the study.

To obtain the variability of the heritability estimates, the weighted bootstrap with 100 weighted datasets was applied (see S3 Text for details). Using 10-fold cross validation, we obtained the average of the complexity parameter values which provided the most regularized model such that the error was within one standard error of the minimum, over 100 weighted datasets. This average was expected to be higher than the complexity parameter of the original data due to the added noise contributed by the weights. We then generated another 100 weighted datasets to obtain the standard error (SE) of the heritability estimates while fixing the complexity parameter value.

In summary, HERRA’s heritability estimate on the observed scale was 0.244 (SE = 1.15 × 10^−2^) and the heritability estimate on the liability scale, assuming the prevalence of CRC equals 0.004, was 0.110 (SE = 5.19 × 10^−3^). In comparison, the GCTA estimate was 0.068 (SE = 0.017) and LDAK estimate was 0.072 (SE = 0.021) [11]. Our proposed estimate was greater than the GCTA and LDAK estimates with smaller standard error, and was closer to heritability estimates from twins and family data, which range from 0.12 to 0.35 [11]. This is probably due to the fact that GWAS SNPs are tagging SNPs and based on our simulation results, the GCTA estimates are biased downward, whereas our proposed estimates generally have a very small empirical bias.

We also evaluated the sensitivity of the choices of the number of iterations and shrinkage parameter values in the screening step on HERRA’s heritability estimation. We varied the ridge shrinkage parameter value from 0.006 to 0.0104, and also iterated the screening for 5 times instead of 6 times within each chromosome. Evidently, the liability-scale heritability estimates were reasonably consistent over a large range of penalty values, as were the iterations (Table 2) except maybe to the case of 5 iterations with shrinkage value of 0.01.

**Table 2:** Heritability estimates in GECCO data: Sensitivity analysis.

Shrinkage	5 Iterations		6 Iterations	
	Observed	Liability	Observed	Liability
0.0060	0.201	0.091	0.207	0.094
0.0080	0.256	0.116	0.236	0.107
0.0100	0.265	0.120	0.244	0.110
0.0102	0.221	0.100	0.218	0.099
0.0104	0.253	0.114	0.257	0.116

Haseman-Elston regression, a popular approach that has recently re-emerged to correct the well-known bias from applying GCTA to ascertained data, is a special case of the Phenotype Correlation—Genotype Correlation (PCGC) of Golan et al. [16]. However, as evident by Fig 2-A and Table 1 of [16], under small values of *h*^2^ (as in our real-data analysis), GREML of GCTA and PCGC are very similar.

### Simulation studies

The following simulation results demonstrate the performance of HERRA and compare it with GCTA and LDAK under various scenarios which include different number of chromosomes and heritability levels. The simulated datasets of one chromosome are based on a phased chromosome 22 of a GWAS study from a colorectal cancer consortium (see the Results section for the details of the consortium), which included 6006 subjects and 9,344 SNPs. Haplotypes were randomly paired to generate the desired sample size *N*. It is assumed that the true models consist of *p* = 100 or 250 randomly chosen causal variants, A setting with *p* = 60 causal variants will be discussed later in the “Additional practical settings” section. The estimation was performed based on *M* = 9,344, or 35,760 observed SNPs when one and five chromosomes were considered, respectively. To simulate data of five chromosomes, we randomly sampled, with replacement, 12,012 haplotypes five times and a total of *p* causal SNPs were randomly selected from a total of 46,720 SNPs. After excluding their corresponding SNPs and their five neighboring SNPs from each side, in the other four chromosomes, the data consisted of 35,760 SNPs. By randomly pairing haplotypes, we generated *N* observations. Since repeatedly running HERRA and GCTA with 35,760 SNPs is time consuming, only limited scenarios are presented for the setting of five chromosomes. Two levels of heritability were considered, *h*^2^ = 0.1 or 0.6, and $\sigma_{e}^{2}=1$ so the total variance explained by the additive genetic effect $\sigma_{g}^{2}=0.111$ or 1.5, respectively. The effects of the standardized causal SNPs, *u*_1_, …, *u*_*p*_, were randomly generated from normal distribution with mean 0 and variance $\sigma_{g}^{2}/p$ such that $\sigma_{g}^{2}=\sum_{j=1}^{p}u_{j}^{2}$. The results are based on 100 random samples for each configuration. S6 Text presents the effect sizes used in the simulation with *p* = 100 causal SNPs and *h*^2^ = 0.1, demonstrating that the simulation settings consist of many small effect sizes.

#### Continuous outcome

Starting with a continuous trait and Model (1) in Methods, we considered the case in which the true model consists of *p* = 100 or 250. With one chromosome of *M* = 9,344 SNPs, the sure independent screening [12] (SIS) as a marginal-type screener method was applied in Step 1 (see Methods). Such correlation learning screens those SNPs that have weak marginal correlations with *Y*. Specifically, we ranked the SNPs according to the magnitude of their sample marginal correlations with the response variable and the top 30% SNPs were kept. Our numerical experience shows that the estimator of $\sigma_{e}^{2}$ is fairly robust to the number of SNPs excluded in Step 1 as long as the sparsity assumption holds. In Steps 2 and 3 (see Methods) only the selected SNPs of Step 1 were used, and lasso regression models were applied with 10-fold cross validation and fixed regularization parameter, chosen based on the average of the first ten samples for saving computational time. Finally, in Step 4 (see Methods) ${\hat{\sigma }}_{e}^{2}$ was calculated.

**Fig 1:**
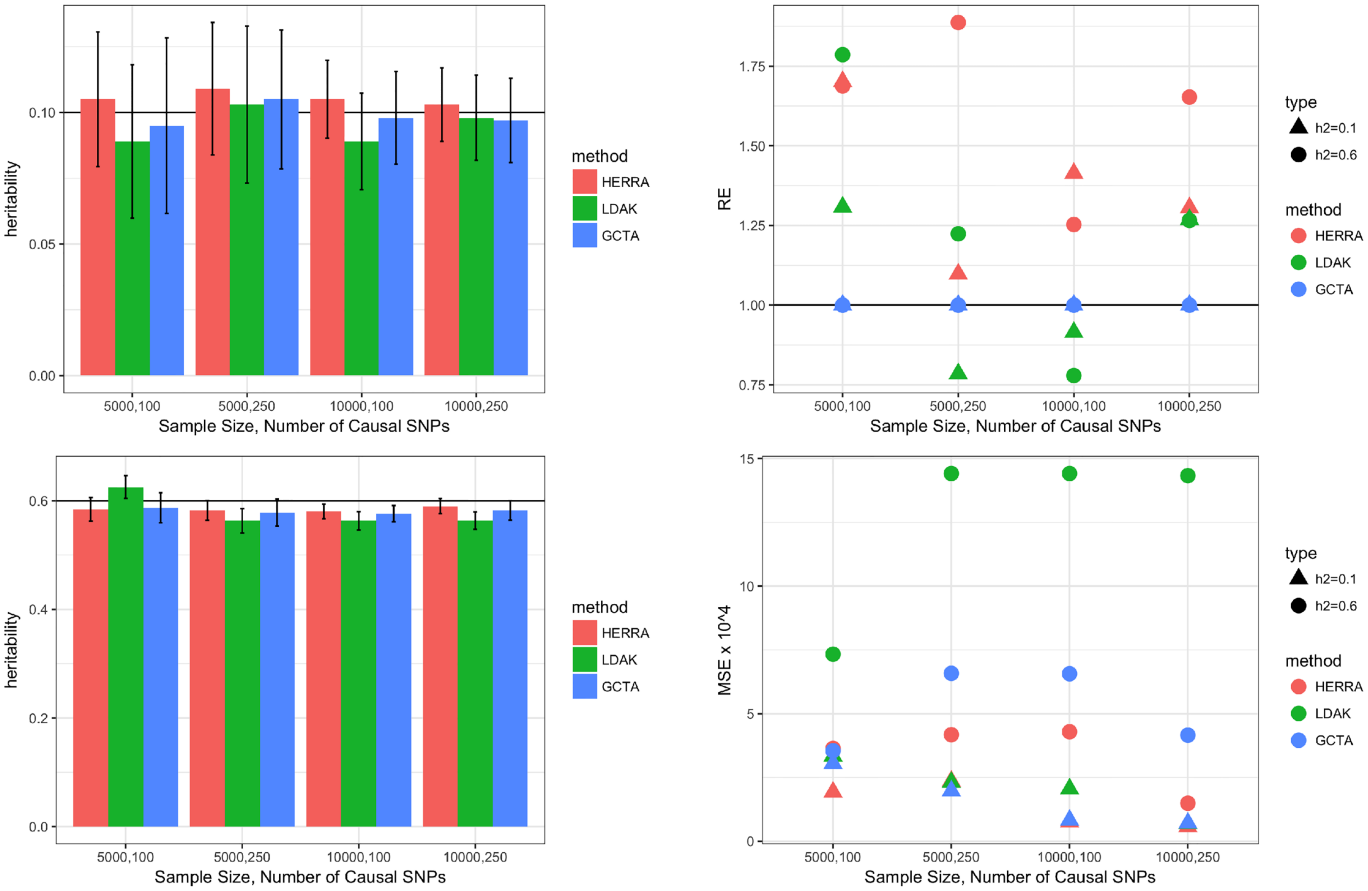
Simulation results of continuous trait and one chromosome: left-top figure is of *h*^2^ = 0.1, left-bottom figure is of *h*^2^ = 0.6; in the left figures colored bar represents the mean of estimated heritability, and vertical black bars are mean ± two standard errors; right figures present the relative efficiency (RE), and mean-squared error (MSE) × 10^4^. For HERRA and LDAK, RE is defined as the ratio of the variance of GCTA’s estimator to the respective variance of HERRA and LDAK estimators.

Fig 1 and S1 Table compare the simulation results of the proposed approach (HERRA), GCTA and LDAK. In the plots of the left column, colored bars present the mean of estimated heritability, and vertical black lines are mean ± two standard errors. The plots of the right column present the relative efficiency (RE) and the mean squared error (MSE) of the estimators. For HERRA or LDAK, RE is defined as the ratio of the variance of GCTA’s estimator to the respective variance of HERRA or LDAK estimators. RE greater (less) than 1 indicates that HERRA or LDAK estimators are more (less) efficient compared to GCTA; i.e., it requires fewer (more) samples than GCTA to achieve a given performance. MSE is defined as the squared bias plus the variance, and minimizing the MSE is a well-known desirable key criterion. Fig 1 suggests that the three methods perform well in terms of bias, while HERRA usually has smaller standard errors and smaller MSEs in estimating *h*^2^, and thus we conclude that HERRA is often more efficient than GCTA or LDAK. S1 Table indicates that HERRA usually has smaller standard errors in estimating $\sigma_{\epsilon }^{2}$. In S1 Text we showed that HERRA is a consistent estimator.

#### Dichotomous outcome

**Fig 2:**
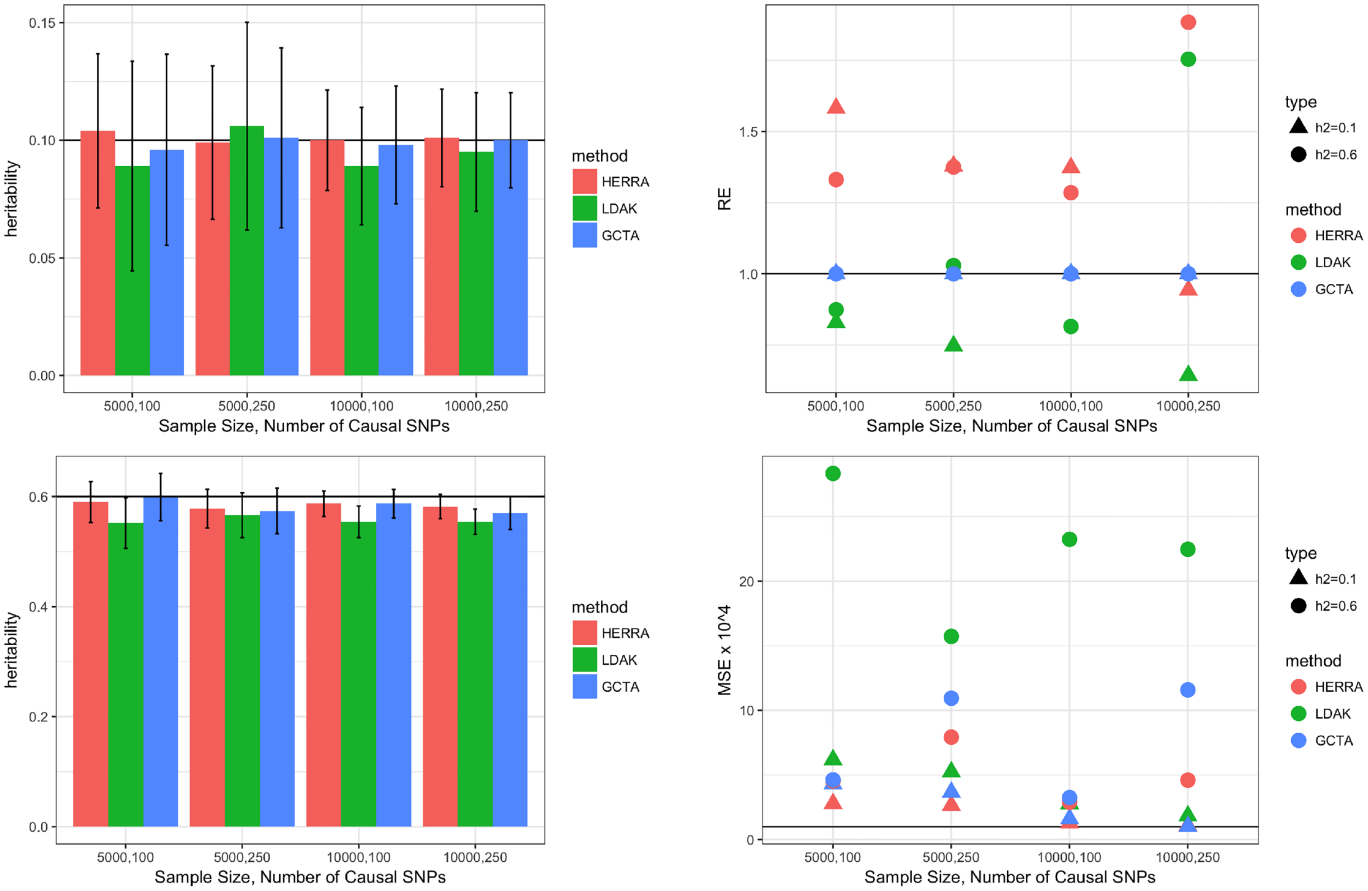
Simulation results of dichotomous trait and one chromosome: left-top figure is of *h*^2^ = 0.1, left-bottom figure is of *h*^2^ = 0.6; in the left figures colored bar represents the mean of estimated heritability, and vertical black bars are mean ± two standard errors; right figures present the relative efficiency (RE), and mean-squared error (MSE) × 10^4^. For HERRA and LDAK, RE is defined as the ratio of the variance of GCTA’s estimator to the respective variance of HERRA and LDAK estimators.

The simulated datasets for a dichotomous trait and Model (2) in Methods were generated in a similar fashion. In particular, a continuous outcome *Y* was first generated as described above, and *D* was set to 1 if *Y* > 0 and 0 otherwise, thus *K* = 0.5. Fig 2 and S2 Table summarize the results of a dichotomous outcome with *K* = 0.5. Similar results were found with other values of *K*, so those results are not shown. Evidently, also for a dichotomous outcome, the three methods perform well in terms of bias, while HERRA usually has smaller standard errors and MSEs in estimating $\sigma_{\epsilon }^{2}$, $h_{o}^{2}$ and $h_{l}^{2}$, and thus we again conclude that HERRA is often more efficient than GCTA or LDAK. In S2 Text we showed that HERRA is a consistent estimator.

#### Additional practical settings

**Fig 3:**
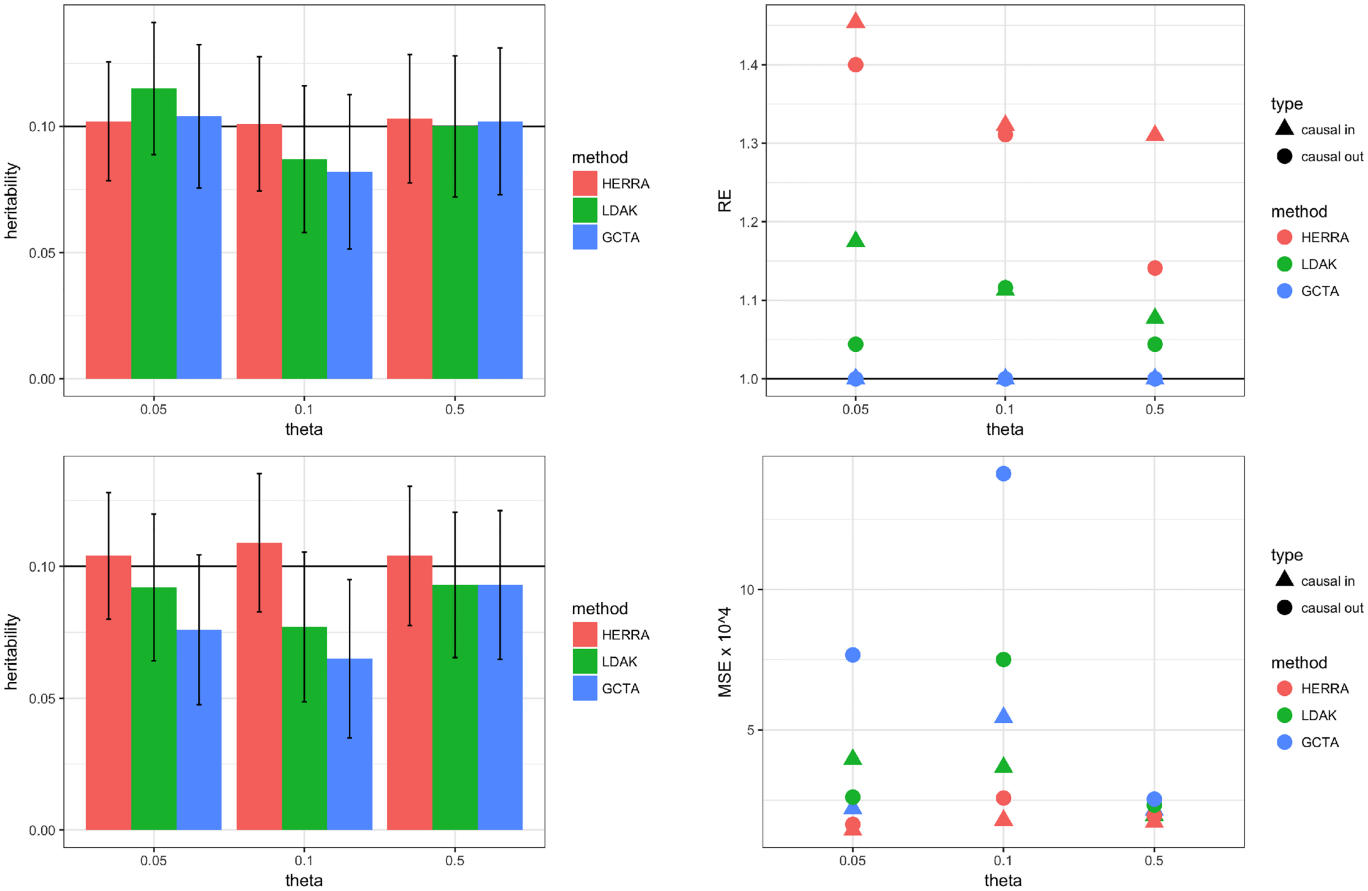
Simulation results of continuous trait, one chromosome, *N* = 5000, *p* = 60, *h*^2^ = 0.1, $\sigma_{e}^{2}=1$, $\sigma_{Y}^{2}=1.111$, $\sigma_{g}^{2}=0.111$, all causal SNPs had a MAF ≤ *θ*: left-top figure presents the results in which all causal SNPs are included in the analysis; in left-bottom figure all the causal SNPs were excluded from the estimating procedure; in the left figures colored bar represents the mean of estimated heritability, and vertical black bars are mean ± two standard errors; right figures present the relative efficiency (RE), and mean-squared error (MSE) × 10^4^. For HERRA and LDAK, RE is defined as the ratio of the variance of GCTA’s estimator to the respective variance of HERRA and LDAK estimators.

Following Yang et al. [3], two additional practically relevant settings were studied: (I) all the causal SNPs have a MAF (denoted by *θ*) less than or equal 0.05 or 0.1; (II) all the causal SNPs are excluded from the estimation procedure, demonstrating the performance of the estimators when the causal variants are not genotyed. Fig 3 and S3 Table summarize the results of the continuous outcome with *p* = 60, *h*^2^ = 0.1 and *N* = 5000. *p* = 60 was chosen due to small number of SNPs with MAF ≤ 0.05. Similar results were observed for *h*^2^ = 0.6 and for the binary outcome setting, and thus those results are not shown. Evidently, the superiority of HERRA is most prominent in Scenario II in which all the causal SNPs were not included in the estimation procedure. In this case, GCTA and LDAK tend to underestimate the true heritability while HERRA shows very small empirical bias. All the three methods, GCTA, LDAK and HERRA exploit the high association between the causal SNPs and their neighboring SNPs, but this is done more successfully by HERRA, due to the screening and variable selection of Steps 1–3 (see Methods). In contrast, GCTA and LDAK estimate similarities but the fact that causal variants are not in the genotyped data makes the estimation of similarities more difficult.

**Fig 4:**
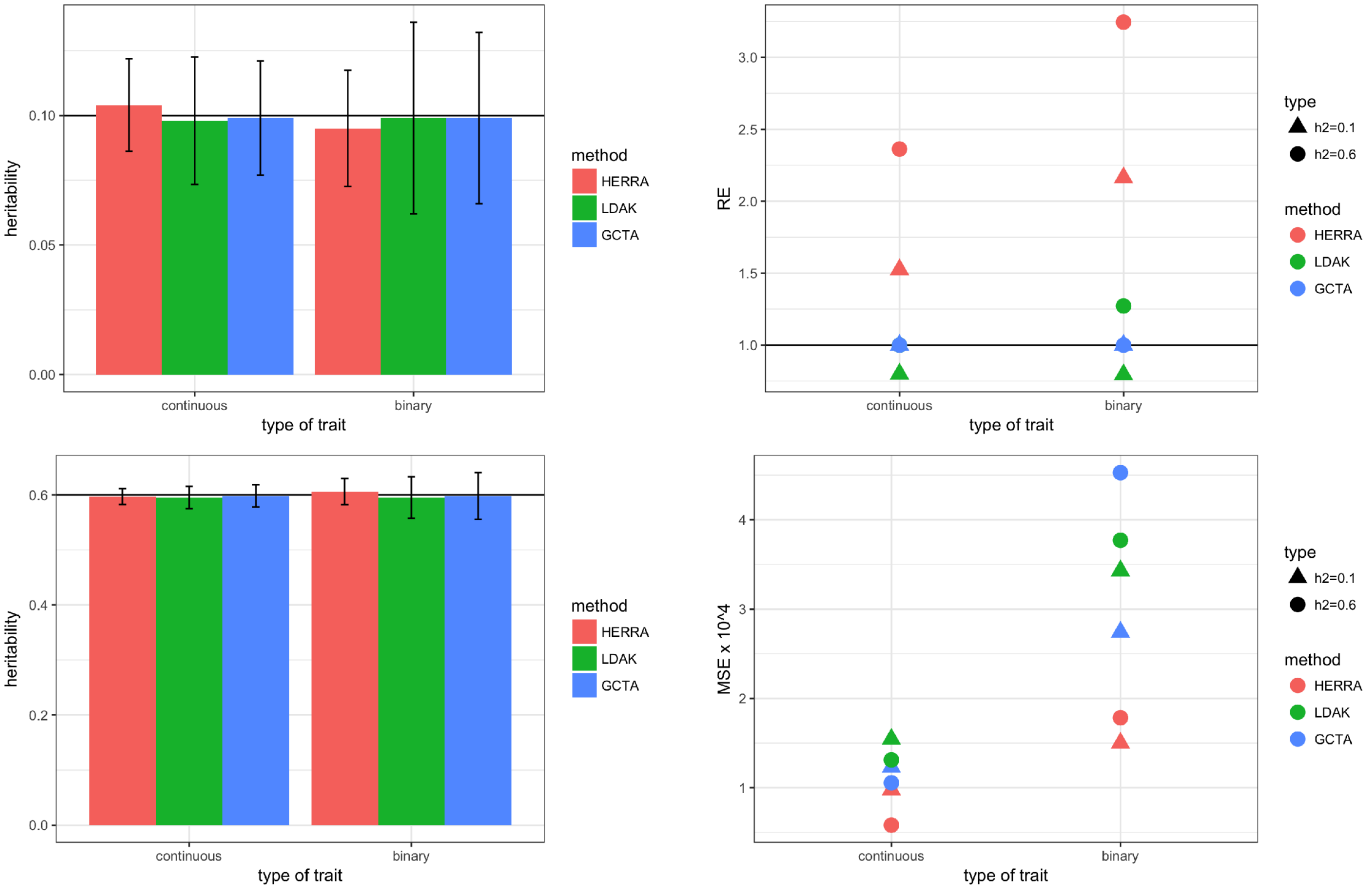
Simulation results of five chromosomes, *M* = 35,760, *N* = 10,000, *p* = 250: left-top figure is of *h*^2^ = 0.1, left-bottom figure is of *h*^2^ = 0.6; in the left figures colored bar represents the mean of estimated heritability, and vertical black bars are mean ± two standard errors; right figures present the relative efficiency (RE), and mean-squared error (MSE) × 10^4^. For HERRA and LDAK, RE is defined as the ratio of the variance of GCTA’s estimator to the respective variance of HERRA and LDAK estimators.

**Fig 5:**
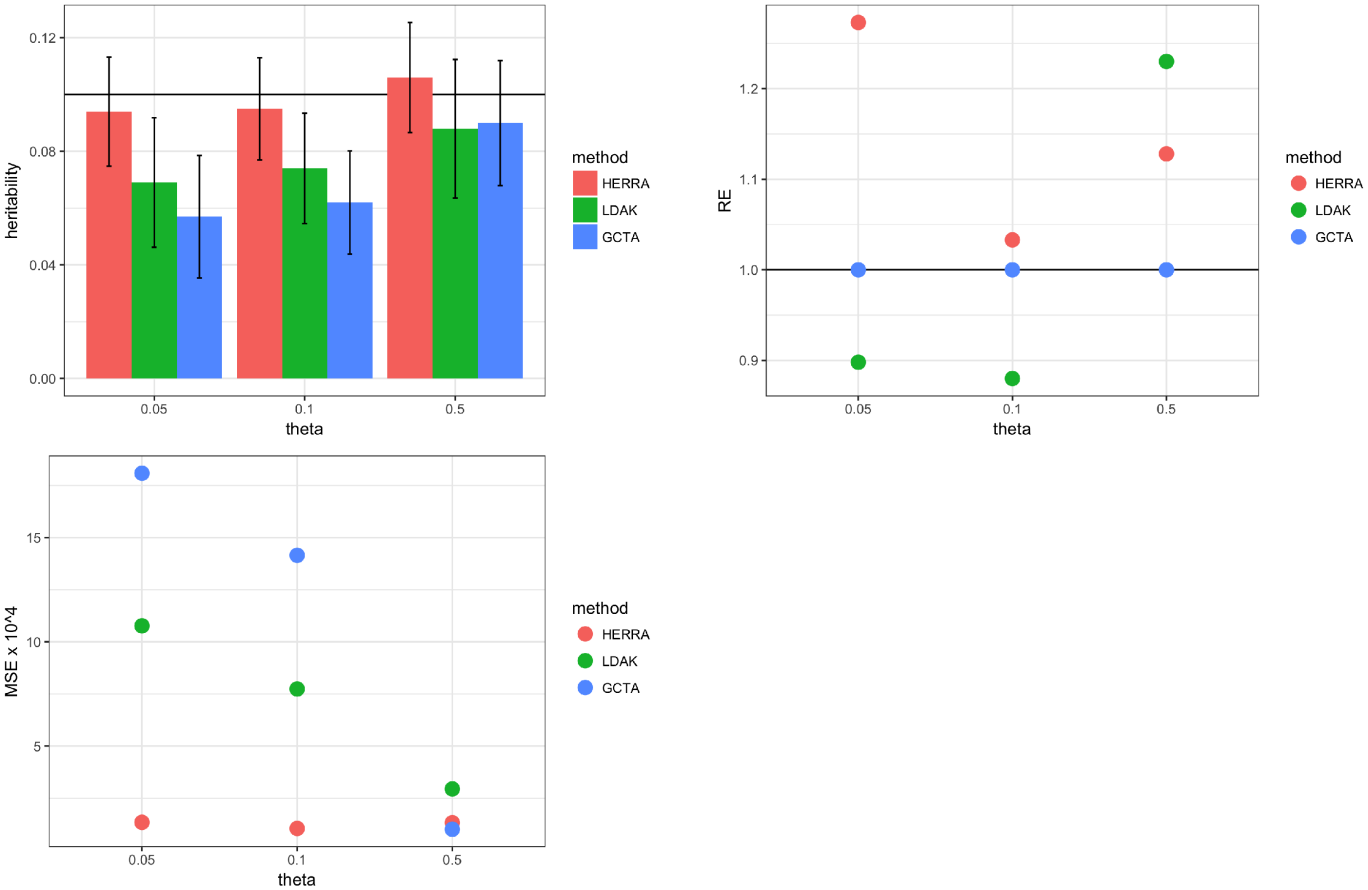
Simulation results of five chromosomes, continuous trait, *h*^2^ = 0.1, *M* = 35,760, *N* = 10,000, *p* = 250, all causal SNPs had a MAF ≤ *θ*: lef-top figure shows means of estimated hertiability that are represented by colored bars, mean ± two standard errors are the vertical black bars; right-top figure presents relative efficiency (RE); left-bottom figure presents the mean-squared error (MSE) × 10^4^. For HERRA and LDAK, RE is defined as the ratio of the variance of GCTA’s estimator to the variance of HERRA’s and LDAK’s estimator, respectively.

Next, we compared HERRA, GCTA and LDAK when the data consisted of five chromosomes. In Step 1 of HERRA’s algorithm, the ITRRS was applied separately for each chromosome, with 5 iterations. In each iteration, the top 50% of the SNPs were selected based on the absolute value of the estimated regression coefficient. Finally, all the selected SNPs of the five chromosomes were combined. The rest of the estimation procedure follows the same steps as in the real data analysis and other simulation settings. Fig 4 and the top of S4 Table summarize the results of the continuous and binary settings where all of the *p* = 250 causal SNPs were genotyped. Fig 5 and the bottom of S4 Table summarize the results of Scenario II, of the continuous trait, in which all the causal SNPs had a MAF less than *θ*, and were excluded at the estimation stage. Again, as long as the causal SNPs are genotyped, the three methods provide heritability estimators with no evidence for empirical bias while HERRA is more efficient. However, under Scenario II, GCTA and LDAK tend to underestimate the heritabilty whereas HERRA still shows very small empirical bias.

The reported simulation results of HERRA are based on selecting the top 50% of the SNPs with largest absolute values of estimated ridge-regression coefficients. Although a smaller subset can be selected (e.g., top 20%) with fewer rounds of ridge regression analyses, we recommend on a moderate cutoff and multiple rounds of ridge regression analysis. This is because the presence of a very large number of non-causal SNPs in the model can cause high variability in the regression coefficient estimates, a graduate dimension-reduction process is preferable. A small-scale sensitivity analysis with various cutoff values, top 40%, 50%, and 60%, five chromosomes, five iterated ridge analyses, *h*^2^ = 0.1, and continuous outcome, yields, ${\hat{h}}^{2}=0.0965$ (SE = 9.57 × 10^−2^), ${\hat{h}}^{2}=0.0991$ (SE = 8.56 × 10^−2^) and ${\hat{h}}^{2}=0.0956$ (SE = 8.08 × 10^−2^), respectively. These estimates are similar, suggesting the estimation is relatively robust against the choice of the percentage of top SNPs being selected.

#### Age-at-onset outcome

Lastly, we studied age-at-onset with right-censored data using the log-normal accelerated failure time (AFT) Model (3) in Methods. Table 3 presents the results of 100 causal SNPs that were randomly selected from five chromosomes. The censoring times were assumed to be zero-mean normally distributed with standard deviation equals 2, yielded 50% censoring rate. Here we considered sample sizes of *N* = 10,000, 15,000 and 20,000, since the presence of censoring requires large sample size. In terms of estimation technique, the main difference between continuous or binary outcomes and age-at-onset with possibly right-censored outcome, is that the heritability estimator for age-at-onset consists of additional weights that are estimated by the data (for details see the Methods). Thus, based on a 10-fold cross validation, the regularization parameter of lasso was chosen to be the one which gives the most regularized model such that error is within one standard error of the minimum. This modification is required due to the added noise contributed by the estimated weights. Since no available heritability estimator can handle right-censored data, Table 3 summarized only the results of HERRA. Evidently, the proposed estimator performs very well in terms of bias. The small empirical bias of HERRA’s estimate observed in the simulation, is along the line of the theoretical results indicate that our proposed estimator is consistent.

**Table 3:** Simulation results of HERRA’s estimator for age-at-onset cohort data, five chromosomes, *M* = 35,760, *p* = 100, 50% censoring rate: Empirical mean and SD × 10^2^.

	*h*^2^		$\sigma_{e}^{2}$		$\sigma_{Y^{o}}^{2}$	
*N*	mean	SD × 10^2^	mean	SD × 10^2^	mean	SD × 10^2^
true values: *h*^2^ = 0.1; $\sigma_{e}^{2}=1.0$; $\sigma_{Y^{o}}^{2}=1.111$						
10000	0.086	6.602	1.009	7.353	1.105	2.467
15000	0.102	4.502	0.994	5.387	1.106	1.868
20000	0.108	4.094	0.987	4.889	1.107	1.843
true values: *h*^2^ = 0.6; $\sigma_{e}^{2}=1.0$; $\sigma_{Y^{o}}^{2}=2.5$						
10000	0.569	3.676	1.055	8.253	2.453	7.206
15000	0.576	2.414	1.039	5.379	2.454	6.312
20000	0.575	1.866	1.045	4.401	2.461	5.395

## Discussion

We provided simple, efficient, and consistent estimators (see Supporting Information for consistency proofs) of the narrow-sense heritability based on GWAS data, for a continuous, categorical or age-at-onset outcome where covariates can be readily incorporated. We showed, by simulation, that HERRA provides essentially unbiased results even if the causal SNPs are not genotyped, in contrast to GCTA’s and LDAK’s estimator. For age-at-onset outcome, we are the first to provide a narrow-sense heritability estimator based on GWAS data of unrelated individuals. The analysis of the case-control GECCO data demonstrates that the heritability estimates of GCTA, LDAK and HERRA could be substantially different.

The current methods in the literature assume that the effect sizes of causal SNPs are independent and identically distributed random variables, and often conveniently the normal distribution is adopted. The working-random-effects assumption is used for simplifying the estimation procedure: instead of estimating the individual causal effect sizes, one needs to estimate only the variance of causal effect sizes. Thus estimation of thousands of parameters is replaced by estimating only one parameter. However, in order to do this, a kinship correlation matrix based on whole genome-wide variants is calculated. While corrections have been made to account for including vastly null markers in this calculation, as shown in our simulation and others’ works [5], in some situations the heritability can be underestimated. In contrast, HERRA assumes a fixed effect model and uses modern machine learning algorithms that have been developed in recent years to explicitly select the variants associated with the phenotype. Instead of estimating individual SNP effects that can be highly variable, we propose to estimate the total sum of squared regression effects, which is shown to provide a more robust and efficient estimator of the heritability comparing to existing random-effects approaches. Furthermore, it also naturally incorporates both known and unknown causal loci. For example, should the known causal loci be treated as fixed or random effects? Treating known loci as fixed effects but other SNPs’ effects as random effects doesn’t seem reasonable as those unknown causal loci may be discovered in the near future. On the other hand, treating all causal SNPs, known or unknown, as random, might wrongly weaken the effect of the known causal ones, as the kinship correlation matrix is constructed based on the known causal SNPs and also SNPs with very weak effect sizes (if any). In contrast, the fixed-effect approach as in our paper, is more appropriate. Known causal loci can naturally be included in the model as fixed effects and will not be included in the SNP selection procedure (e.g., ridge and lasso).

HERRA uses dimension reduction methods to deal with the large number of SNPs. With dimensionality reduction from large or huge scale (i.e. exp(*O*(*N*^*c*^)), for some *c* > 0) to a relatively large scale (i.e. *o*(*N*)), an accurate estimator is obtained by using well-developed lower dimensional methods. With a relatively small number of SNPs (e.g. 10,000) we showed that SIS provides a useful screener procedure. However, for a larger number of SNPs (e.g. 35,000 as in the simulations or 250,000 as in the GECCO data) we would recommend the ITRRS as a screener. For example, under a binary outcome, $h_{l}^{2}=0.1$, *M* ≈ 40,000 and *p* = 250, the estimated narrow-sense heritability on liability scale by HERRA with ITRRS and SIS are 0.095 (SE = 0.12 × 10^2^) and 0.224 (SE = 1.26 × 10^2^), respectively. Well-known challenges with high dimensionality are that causal SNPs can be highly correlated with non-causal SNPs and the number of spurious correlations grows with dimensionality. Hence, with such a high number of SNPs, the top SNPs selected by SIS are overloaded with spurious correlated SNPs because SIS, as a marginal association screener, does not account for the correlation among the SNPs.

Screening or variable selection in high- or ultra-high-dimensional methods is very complex. Each method, such as SIS, ITRRS and lasso, is associated with tuning parameter(s) that need to be determined or estimated. The tuning parameters control the amount of regularization and therefore their values may affect the final results. Specifically, SIS requires the determination of a threshold value so that *d* > 0 top ranked covariates are selected. In ITRRS, the number of iterations should be determined, and within each iteration the regularization parameter of the ridge regression model should be chosen. Lasso also requires estimation of the regularization parameter. Estimation of the optimal value of the regularization parameter is often done by one of the following methods: Akaike information criterion (AIC), the Bayes Information criterion (BIC), and cross validation. Each of these methods has its own pros and cons, and not one of them is considered the best approach in general. Moreover, what might be considered as a good choice of tuning parameter depends on whether the goal is prediction accuracy (closer to our interest) or recovering the true model for interpretation purposes (not in our current interest). Based on our extensive numerical experience, choosing between AIC, BIC, and cross validation seems to be obvious as often only one of them keeps a reasonable number of SNPs (for example, AIC keeps hundreds of SNPs while BIC and cross validation keep less than 100). For many complex diseases that have a genetic component, we often have some sense of the ball park number of causal SNPs involved in the disease etiology [17]. Therefore, our practical recommendation is to use threshold values that well accommodate the sparsity assumption. For example, in the GECCO application, we started with 248,977 SNPs, so given the sparsity assumption, the median of the ridge-regression coefficients within each chromosome equals zero. Therefore, no bias is introduced by the dimensionality reduction step. In general, we strongly recommend on performing a sensitivity analysis, as presented in the GECCO data analysis.

Obviously, in practice, by using variable selection techniques such as SIS, ITRRS and lasso, not all the causal SNPs are retained in the selected and instead well-tagging neighboring SNPs are being selected. However, since our aim of the variable selection step is estimating the environmental-effect variance, $\sigma_{e}^{2}$, and not identifying the causal SNPs, this selection step is not introducing asymptotic bias, as showed by [13] and verified in our simulation study. Therefore, HERRA’s heritability estimators converges to the true parameters’ values.

Applying HERRA in age-at-onset outcome requires large sample size due to censoring. For estimating $\sigma_{Y^{o}}^{2}$ consistently, it is assumed that the support of the failure time, *Y*^*o*^, will be covered by the support of the censoring time, *C*. The IPCW-type estimator is consistent if the weight is correctly specified. In case the censoring distribution depends on some covariates, a model (e.g. Cox, AFT) that accommodates this dependency must be correctly specified in the weight estimation stage. However, our heritability estimator of age-at-onset data can be easily applied using the regularized rank-based estimation procedure with Lasso-type penalty [18] and avoid estimating the censored survival function.

Although genome-wide association studies (GWASs) have resulted in the discovery of thousands of variants associated with common diseases and traits, these variants explain only a small portion of the heritability [19]. This has been called the missing heritability problem [20]. For instance, the heritability of human height is about 80% [1, 21] but the ∼700 published SNPs identified from GWAS as associated with height explain only about 20% of the total variance of height [22]; and based on all genotyped SNPs, narrow-sense heritability was estimated to be 45% [2, 6]. Various hypotheses have been proposed for explaining the missing heritability: the existence of many presently unidentified common variants with small effect sizes; some of the causal loci not being in perfect linkage disequilibrium (LD) with the underlying functional SNPs; rare variants not captured by current genotyping platforms; missing epistatic interaction in the model; missing gene-environment interaction in the model; parent-of-origin effect; or inflated heritability estimates based on blood related individuals such as monozygotic and dizygotic twins [4, 23–30]. In this paper we presented a useful methodology for heritability estimation that can be directly extended to include epistatic and gene-environment interactions, as will be presented in future communications.

## Methods

### Continuous outcome

Let *Y*_*i*_ be the continuous phenotype of subject *i* such that

$$Y_{i}=\mu +\sum_{j=1}^{p}X_{ij}u_{j}+e_{i}=\mu +X_{i}^{T}u+e_{i}\;\;\;\;i=1,\ldots ,N$$ (1)

where $X_{i}^{T}=\left(X_{i1},\ldots ,X_{ip}\right)$ is the vector of genotypes of subject *i*, *p* is the total number of latent trait-associated variants, *X*_*ij*_ is the standardized genotype of individual *i* at the *j*th diallelic causal variant given an additive coding of genotypes. Therefore, E(*X*_*ij*_) = 0, var(*X*_*ij*_) = 1, *j* = 1…, *p*, **E** and var denote expectation and variance, respectively. Also, **u**^*T*^ = (*u*_1_, ⋯, *u*_*p*_), *u*_*j*_ is the *j*-th variant regression coefficient, *e*_*i*_, *i* = 1,…, *N*, are independent environmental random effects assumed to follow a normal distribution with mean 0 and variance $\sigma_{e}^{2}$. *μ*, $\sigma_{e}^{2}$ and **u** are unknown parameters. A common working independence assumption among *X*_*ij*_, for all *i* and *j* [31], yields that the total variance explained by the additive genetic effect equals $\sigma_{g}^{2}=\sum_{j=1}^{p}u_{j}^{2}$. Our main concern is estimating

$$h^{2}=\sigma_{g}^{2}/\left(\sigma_{g}^{2}+\sigma_{e}^{2}\right),$$

the proportion of the total variance explained by the additive genetic effect. The most popular estimators of *h*^2^ are based on plugging in a type of GREML estimator of $\left(\sigma_{g}^{2},\sigma_{e}^{2}\right)$ [2–6]. Specifically, the GCTA software [3] estimates heritability by treating *u*_1_, …, *u*_*p*_ as zero-mean independent normally distributed random variables with variance $\sigma_{g}^{2}/p$, and estimates $\sigma_{g}^{2}$ directly (not through $\sum_{j=1}^{p}u_{j}^{2}$). Additionally, the GREML-type estimators use several crucial calibrating steps. Since the *p* trait-associated variants are unknown, GCTA uses all the *M* ≫ *p* observed SNPs from dense GWAS data for estimating the similarities between individuals. However, the heritability estimator may become unstable if the proportion of causal variants is low due to the large number of non-causal variants that mask the true similarities [5, 6]. This concern motivates us to consider an alternative approach: First, we select variants by using modern regularized regression techniques, and then estimate heritability using the selected variants, as elaborated in the following section.

The phenotypic variance equals $\sigma_{Y}^{2}=\sigma_{g}^{2}+\sigma_{e}^{2}$. However, estimating heritability based on estimating $\left(\sigma_{g}^{2},\sigma_{e}^{2}\right)$, as in GCTA, is not the same as estimating heritability based on estimating $\left(\sigma_{Y}^{2},\sigma_{e}^{2}\right)$, since the identity of the *p* causal SNPs is unknown which requires estimating the variance components based on a working model instead of the correct Model 1. Our novel approach is to estimate heritability by estimating $\left(\sigma_{Y}^{2},\sigma_{e}^{2}\right)$ and then to use the identity

$$\begin{array}{c} h^{2}=1-\sigma_{e}^{2}/\sigma_{Y}^{2}. \end{array}$$

The reason for estimating $\sigma_{Y}^{2}$ instead of $\sigma_{g}^{2}$ is that in finite sample sizes, the large number of variants and the LD among variants can cause unreliable estimates of the regression coefficients {*u*_*j*_} and hence of $\sigma_{g}^{2}=\sum_{j=1}^{p}u_{j}^{2}$. In contrast, $\sigma_{Y}^{2}$ can be simply and reliably estimated by the usual unbiased empirical variance estimator ${\hat{\sigma }}_{Y}^{2}=\sum_{i=1}^{N}{\left(Y_{i}-\bar{Y}\right)}^{2}/\left(N-1\right)$, $\bar{Y}=\sum_{i=1}^{N}Y_{i}/N$. A stable consistent estimator of $\sigma_{e}^{2}$ based on *M* observed SNPs from GWAS data is a refitted cross-validation variance estimator in the spirit of Fan et al. [13] that can handle high or ultrahigh dimensions. In particular, we propose the following algorithm:

Step 1 Apply a joint-type screening method such as the iteratively thresholded ridge regression screener [12] (ITRRS) or a marginal-type sure independent screening [12] (SIS) and reduce the ultra-high dimensionality to a relatively large scale but below the sample size *N*. This step is to filter out SNPs that are unlikely to be associated with the trait.
Step 2 Use only those SNPs that are selected in the screening stage of Step 1, and randomly split the sample into two equals subsets. Then, apply a high-dimensional variable selection method, such as lasso, to the first subset, yielding a parsimonious model with a small set of selected SNPs. Apply the ordinary least-squares method to the second subset using only the selected SNPs to obtain unbiased estimates of the regression coefficients and $\sigma_{e}^{2}$.
Step 3 Repeat Step 2 while switching the role of the first and second subsets.
Step 4 The final estimator of $\sigma_{e}^{2}$ is defined as the mean of the above two estimators obtained in Steps 2 and 3, denoted by ${\hat{\sigma }}_{e}^{2}$.

Finally, our simple consistent heritability estimator is defined as

$$\begin{array}{c} {\hat{h}}^{2}=1-{\hat{\sigma }}_{e}^{2}/{\hat{\sigma }}_{Y}^{2}. \end{array}$$

Based on our extensive simulation study, we conclude that a marginal-type screening technique such as SIS could provide good results in terms of bias, as long as the number of SNPs involved is not large, e.g., 10,000. For higher numbers of SNPs, a joint-type screener, such as the ITRRS, should be used so that the LD between the SNPs is considered and the truly associated SNPs can be better selected.

Our procedure can be modified to estimate heritability to account for known factors such as smoking and dietary variables, as is often of interest in practice, to account for the confounding effect and reduce the error variance so that the additive genetic effects in the heritability can be more accurately estimated. Specifically, the known risk factors **W** are included in the model and will not be subject to variable selection, either in Step 1, or in Step 2. Then the heritability estimator accounting for risk factors **W** is defined by $1-\left\{{\hat{\sigma }}_{e}^{2}+{\hat{\beta }}^{T}\hat{\text{var}}\left(W\right)\hat{\beta }\right\}/{\hat{\sigma }}_{Y}^{2}$, where $\hat{\beta }$ is the regression coefficient estimator of **W**.

Since the lasso possesses the oracle property, it can be shown (details in S1 and S2 Text) that, as the sample size increases, ${\hat{h}}^{2}$ converges to the true heritability value, i.e., ${\hat{h}}^{2}$ is a consistent estimator. In addition, the asymptotic variance of $\sqrt{N}{\hat{h}}^{2}$ equals 4*h*^2^(1 − *h*^2^)^2^. Although the oracle property of the lasso estimators allows one to carry out statistical inference on the nonzero regression parameters and *h*^2^, following variable selection, the accuracy of the resulting inference remains unknown [32]. Alternatively, we proposed a weighted bootstrap variance estimator, and based on the oracle property of ${\hat{\sigma }}_{e}^{2}$, Step 1 of the estimation procedure need not be included in the bootstrap procedure (see S3 Text for details).

### Categorical outcome

The heritability of all-or-none (0/1) traits, such as disease status, can be defined, as for the continuous outcome, as the proportion of variation that is due to additive genetic factors. However, variances and heritabilty calculated on an observed scale, for example, 0 or 1, are functions of the prevalence of the trait in the population [33, 34]. Wright [35] suggested that all-or-none traits can be represented by an underlying normally-distributed liability trait. Namely, as described by Falconer [36], we assume there is in fact an underlying gradation of some attribute immediately related to the causation of the disease. If we could measure this attribute, it would give us a graded scale of the degree of affectedness or of normality, and we would find that all individuals above a certain value exhibited the disease and all below it did not. This hypothetical graded attribute is referred to as the individual’s liability for the disease. A liability trait, as for a continuous trait, is defined as the sum of independent normally-distributed genetic and environmental components [14, 36]. The advantage of the liability scale is that heritability is independent of prevalence and can therefore be compared across traits or populations.

In recent works [6, 15] the all-or-none trait was expressed as a linear function of the sum of the additive effects due to SNPs associated with causal variants and homoscedastic normally-distributed residual effect. Based on the random-effects model approach, the variance components of the model were estimated using a type of GREML method, and the resulting heritability estimator was on the observed scale. Then, the Robertson transformation [14] was applied yielding a heritability estimator on the liability scale. However, we proposed a simpler and more efficient estimator where the random-effects approach is replaced by a regularized regression approach, as described below.

Let *D*_*i*_, *i* = 1, …, *N*, be a binary outcome and consider the linear working model of the form

$$\begin{array}{c} D_{i}=\alpha +X_{i}^{T}v+\epsilon_{i}, \end{array}$$ (2)

where **v**^*T*^ = (*v*_1_, ⋯, *v*_*p*_), *v*_*j*_ is the variant’s regression coefficient of causal SNP *j*, $\sigma_{og}^{2}=\sum_{j=1}^{p}v_{j}^{2}$, *ϵ*_*i*_, *i* = 1, …, *N*, are independent random zero-mean normally-distributed variables with variance $\sigma_{\epsilon }^{2}$, and *α*, **v** and $\sigma_{\epsilon }^{2}$ are unknown parameters. First we estimate $h_{o}^{2}=1-\sigma_{\epsilon }^{2}/\sigma_{D}^{2}$, and then, by the Robertson transformation [14] we get heritability in liability scale, $h_{l}^{2}$.

We start by estimating $\sigma_{\epsilon }^{2}$ by ${\hat{\sigma }}_{\epsilon }^{2}$, based on Steps 1—Step 4 above. The estimator of the total variance of the binary outcome is defined as ${\hat{\sigma }}_{D}^{2}=\bar{D}\left(1-\bar{D}\right)$, where $\bar{D}=\sum_{i=1}^{N}D_{i}/N$, and finally, the proposed heritability estimator in the observed 0/1 scale is defined as ${\hat{h}}_{o}^{2}=1-{\hat{\sigma }}_{\epsilon }^{2}/{\hat{\sigma }}_{D}^{2}$. Applying the Robertson transformation yields a heritability estimator on the liability scale ${\hat{h}}_{l}^{2}={\hat{h}}_{o}^{2}K\left(1-K\right)/z^{2}$, where *z* is the height of the standard normal curve at the threshold that truncates the proportion *K*. In S2 Text, we show that ${\hat{h}}_{l}^{2}$ is a consistent estimator of the true heritability on liability scale. To account for known risk factors, **W**, the modification described in the continuous setting, applies here as well. The variance of ${\hat{h}}_{l}^{2}$ is estimated by the weighted bootstrap approach (see S3 Text for details).

### Age-at-onset outcome

Consider the following popular parametric accelerated failure time model

$$Y_{i}^{o}=\mu +X_{ij}^{T}u+e_{i}\;\;\;\;i=1,\ldots ,N$$ (3)

where $Y_{i}^{o}=\log T_{i}$, *T*_*i*_ is the failure-time random variable, and the *e*_*i*_s are independent normally distributed with mean zero and variance $\sigma_{e}^{2}$. The log-scale censoring times *C*_*i*_, *i* = 1, …, *N*, are assumed independent and identically distributed. The log-scale observed times are then $Y_{i}=\min\left(Y_{i}^{o},C_{i}\right)$ and the event indicators are defined by *δ*_*i*_ = *I*(*Y*_*i*_ ≤ *C*_*i*_), *i* = 1, …, *N*. Hence the available data can be summarized by $\left\{Y_{i},X_{i}^{T},\delta_{i}\right\}$, *i* = 1, …, *N*, independent observations. Our goal is estimating $h^{2}=1-\sigma_{e}^{2}/\sigma_{Y^{o}}^{2}$.

By adopting the inverse probability censoring weighting (IPCW) approach, the strategy of heritability estimation of continuous trait can be used here as well, with several modifications. Specifically, let ${\hat{S}}_{c}\left(\cdot \right)$ be the Kaplan-Meier estimator of the censoring survival distribution and define the weights $W_{i}=\delta_{i}/{\hat{S}}_{c}\left(Y_{i}\right)$, *i* = 1, …, *N*. Then, $\sigma_{e}^{2}$ is estimated by Steps 1–4 while using weighted linear least squares with the IPCW weights *W*_1_, …, *W*_*N*_. Finally, $\sigma_{e}^{2}$ is estimated by

$$\begin{array}{c} {\hat{\sigma }}_{Y^{o}}^{2}=V_{1}\sum_{i=1}^{n}W_{i}{\left(Y_{i}-{\bar{Y}}_{w}\right)}^{2}/\left(V_{2}-V_{1}\right), \end{array}$$

where ${\bar{Y}}_{w}=\sum_{i=1}^{n}W_{i}Y_{i}/V_{1}$, $V_{1}=\sum_{i=1}^{n}W_{i}$, and with $V_{2}=\sum_{i=1}^{n}W_{i}^{2}$. The consistency of ${\hat{\sigma }}_{e}^{2}$ and ${\hat{\sigma }}_{Y^{o}}^{2}$ to the true variances hold due to the consistency property of the Kaplan-Meier estimator. Thus, the consistency proof of ${\hat{h}}^{2}=1-{\hat{\sigma }}_{e}^{2}/{\hat{\sigma }}_{Y^{o}}^{2}$ is similar to that of continuous outcome.

## Supporting information

S1 Table Tables of simulation results. Details of simulation results that are summarized by figures in the main text—continuous trait, one chromosome.
(PDF)

S2 Table Tables of simulation results. Details of simulation results that are summarized by figures in the main text—dichotomous trait, one chromosome.
(PDF)

S3 Table Tables of simulation results. Details of simulation results that are summarized by figures in the main text—continuous trait, one chromosome, Scenarios I and II.
(PDF)

S4 Table Tables of simulation results. Details of simulation results that are summarized by figures in the main text—five chromosomes.
(PDF)

S1 Text Consistency for continuous outcome. A proof of consistency of HERRA estimator for continuous outcome is sketched, along with the asymptotic distribution of the estimator.
(PDF)

S2 Text Consistency for dichotomous outcome. A proof of consistency of HERRA estimator for dichotomous outcome is presented in details.
(PDF)

S3 Text Weighted-bootstrap variance estimator. The weighted bootstrap variance estimator is described in details.
(PDF)

S4 Text The study population. A Description of the study populations included in Genome-wide Association Study Analysis (GWAS) is detailed.
(PDF)

S5 Text The study population. Details on quality assurance and quality control of the colorectal cancer GWAS dataset.
(PDF)

S6 Text Effect sizes. Effect sizes used in the simulations with *p* = 100 causal SNPs and *h*^2^ = 0.1.
(PDF)

S7 Text Funding and acknowledgments. Detailed funding and cknowledgments list.
(PDF)
