# Supplementary material for: Heritability Estimation using a Regularized Regression Approach (HERRA): Applicable to continuous, dichotomous or age-at-onset outcome
Source: PLoS One. 2017 Aug 16;12(8):e0181269. doi: 10.1371/journal.pone.0181269 (PMC5559077; doi:10.1371/journal.pone.0181269)
Supplement: S1 Table — Details of simulation results that are summarized by figures in the main text—continuous trait, one chromosome. (PDF) [file pone.0181269.s001.pdf]

Heritability Estimation using a Regularized Regression Approach (HERRA): Applicable to Continuous, Dichotomous or Survival Outcome

Malka Gorfine[1,*], Sonja I Berndt[2], Jenny Chang-Claude[3], Michael Hoffmeister[4], Loic Le Marchand[5], John Potter[6], Martha L Slattery[7], Nir Keret[1], Ulrike Peters[6], Li Hsu[6,*]

1 Department of Statistics and Operation Research, Tel Aviv University, Tel Aviv, Israel

2 Division of Cancer Epidemiology and Genetics, National Cancer Institute, National Institutes of Health

3 Division of Cancer Epidemiology, German Cancer Research Center, Heidelberg, Germany

4 Division of Clinical Epidemiology and Aging Research, German Cancer Research Center, Heidelberg, Germany

5 Epidemiology Program, University of Hawaii Cancer Center

6 Public Health Sciences Division, Fred Hutchinson Cancer Research Center, Seattle, WA

7 Department of Internal Medicine, University of Utah Health Sciences Center

*** Correspondence: gorfinem@post.tau.ac.il, lih@fredhutch.org**

S1 Table: Tables of simulation results

The following S1 Table provides details of simulation results that are summarized by figures in the main text - continuous trait, one chromosome.

Table S1. Simulation results of continuous trait and one chromosome: empirical mean (empirical SD$\times 10^2$), relative efficiency (RE), and mean-squared error (MSE)$\times 10^4$. For HERRA, RE is defined as the ratio of the variance of GCTA's estimator to the variance of HERRA's estimator. RE greater than 1 indicates that HERRA's estimator is more efficient. p equals the number of causal SNPs.

		h^2			σ_e^2	σ_Y^2	σ_g^2
p		mean (SD)	RE	MSE	mean (SD)	mean (SD)	mean (SD)
true values: $h^2 = 0.1$; $\sigma_e^2 = 1$; $\sigma_Y^2 = 1.111$; $\sigma_g^2 = 0.111$							
$N = 5000$							
100	H	0.105 (1.28)	1.702	1.930	0.986 (2.08)	1.102 (2.17)	-
	L	0.089 (1.46)	1.308	3.342	1.004 (2.37)	-	0.098 (1.65)
	G	0.095 (1.67)	1.000	3.049	0.998 (2.46)	-	0.105 (1.88)
250	H	0.109 (1.26)	1.098	2.357	0.993 (2.15)	1.109 (2.32)	-
	L	0.103 (1.49)	0.785	2.310	0.999 (2.23)	-	0.115 (1.17)
	G	0.105 (1.32)	1.000	1.980	0.996 (2.21)	-	0.117 (1.53)
$N = 10000$							
100	H	0.105 (0.74)	1.414	0.759	0.988 (1.41)	1.104 (1.61)	-
	L	0.089 (0.92)	0.915	2.056	1.005 (1.52)	-	0.098 (1.06)
	G	0.098 (0.88)	1.000	0.827	0.996 (1.51)	-	0.108 (1.02)
250	H	0.103 (0.70)	1.306	0.574	0.996 (1.46)	1.110 (1.51)	-
	L	0.098 (0.81)	1.269	0.696	0.999 (1.53)	-	0.109 (0.93)
	G	0.097 (0.80)	1.000	0.713	0.999 (1.54)	-	0.108 (0.92)
true values: $h^2 = 0.6$; $\sigma_e^2 = 1$; $\sigma_Y^2 = 2.5$; $\sigma_g^2 = 1.5$							
$N = 5000$							
100	H	0.584 (1.07)	1.688	3.641	0.998 (2.26)	2.400 (4.80)	-
	L	0.625 (1.04)	1.786	7.332	0.989 (2.58)	-	1.649 (0.049)
	G	0.587 (1.39)	1.000	3.545	0.978 (2.65)	-	1.393 (5.70)
250	H	0.582 (0.91)	1.887	4.177	1.041 (2.46)	2.489 (5.04)	-
	L	0.563 (1.13)	1.224	14.967	1.047 (2.87)	-	1.349 (4.25)
	G	0.578 (1.25)	1.000	6.580	1.016 (2.86)	-	1.390 (4.87)
$N = 10000$							
100	H	0.580 (0.67)	1.253	4.291	1.008 (1.50)	2.403 (3.71)	-
	L	0.563 (0.85)	0.779	14.413	1.061 (1.18)	-	1.367 (3.45)
	G	0.576 (0.75)	1.000	6.565	0.992 (1.63)	-	1.345 (3.14)
250	H	0.590 (0.70)	1.653	1.490	1.021 (1.69)	2.491 (3.19)	-
	L	0.563 (0.80)	1.266	14.330	1.047 (1.77)	-	1.351 (3.28)
	G	0.582 (0.90)	1.000	4.159	1.012 (1.73)	-	1.407 (4.05)

H - HERRA, L - LDAK, G - GCTA
