# Supplementary material for: Heritability Estimation using a Regularized Regression Approach (HERRA): Applicable to continuous, dichotomous or age-at-onset outcome
Source: PLoS One. 2017 Aug 16;12(8):e0181269. doi: 10.1371/journal.pone.0181269 (PMC5559077; doi:10.1371/journal.pone.0181269)
Supplement: S2 Table — Details of simulation results that are summarized by figures in the main text—dichotomous trait, one chromosome. (PDF) [file pone.0181269.s002.pdf]

Heritability Estimation using a Regularized Regression Approach (HERRA): Applicable to Continuous, Dichotomous or Survival Outcome

Malka Gorfine[1,*], Sonja I Berndt[2], Jenny Chang-Claude[3], Michael Hoffmeister[4], Loic Le Marchand[5], John Potter[6], Martha L Slattery[7], Nir Keret[1], Ulrike Peters[6], Li Hsu[6,*]

1 Department of Statistics and Operation Research, Tel Aviv University, Tel Aviv, Israel

2 Division of Cancer Epidemiology and Genetics, National Cancer Institute, National Institutes of Health

3 Division of Cancer Epidemiology, German Cancer Research Center, Heidelberg, Germany

4 Division of Clinical Epidemiology and Aging Research, German Cancer Research Center, Heidelberg, Germany

5 Epidemiology Program, University of Hawaii Cancer Center

6 Public Health Sciences Division, Fred Hutchinson Cancer Research Center, Seattle, WA

7 Department of Internal Medicine, University of Utah Health Sciences Center

*** Correspondence: gorfinem@post.tau.ac.il, lih@fredhutch.org**

S2 Table: Tables of simulation results

The following S2 Table provides details of simulation results that are summarized by figures in the main text - dichotomous trait, one chromosome.

Table S1. Simulation results of dichotimous trait and one chromosome: empirical mean (empirical SD$\times 10^2$), relative efficiency (RE), and mean-squared error (MSE)$\times 10^4$. For HERRA, RE is defined as the ratio of the variance of GCTA's estimator to the variance of HERRA's estimator. RE greater than 1 indicates that HERRA's estimator is more efficient. p equals the number of causal SNPs.

		h_l^2			σ_ϵ^2	σ_D^2	σ_{og}^2
p		mean (SD)	RE	MSE	mean (SD)	mean (SD)	mean (SD)
true values: $h_l^2 = 0.1$; $h_o^2 = 0.064$; $\sigma_\epsilon^2 = 0.234$; $\sigma_D^2 = 0.25$; $\sigma_{og}^2 = 0.016$							
$N = 5000$							
100	H	0.104 (1.64)	1.582	2.787	0.233 (0.26)	0.250 (0.006)	-
	L	0.089 (2.23)	0.829	6.183	0.236 (0.35)	-	0.014 (0.35)
	G	0.096 (2.03)	1.000	4.324	0.235 (0.32)	-	0.015 (0.32)
250	H	0.099 (1.63)	1.379	2.650	0.234 (0.26)	0.250 (0.007)	-
	L	0.106 (2.21)	0.747	5.244	0.233 (3.55)	-	0.017 (0.35)
	G	0.101 (1.91)	1.000	3.668	0.233 (0.31)	-	0.017 (0.30)
$N = 10000$							
100	H	0.100 (1.07)	1.373	1.303	0.234 (0.17)	0.250 (0.004)	-
	L	0.089 (1.25)	1.000	2.773	0.236 (1.98)	-	0.014 (0.19)
	G	0.098 (1.25)	1.000	1.611	0.235 (0.19)	-	0.016 (0.20)
250	H	0.101 (1.04)	0.943	1.083	0.234 (0.16)	0.250 (0.004)	-
	L	0.095 (1.26)	0.643	1.838	0.235 (0.20)	-	0.015 (0.20)
	G	0.100 (1.01)	1.000	1.020	0.234 (0.17)	-	0.016 (0.16)
true values: $h_l^2 = 0.6$; $h_o^2 = 0.382$; $\sigma_\epsilon^2 = 0.154$; $\sigma_D^2 = 0.25$; $\sigma_{og}^2 = 0.095$							
$N = 5000$							
100	H	0.590 (1.86)	1.331	4.538	0.156 (0.30)	0.250 (0.004)	-
	L	0.552 (2.30)	0.874	28.330	0.162 (0.36)	-	0.088 (0.37)
	G	0.599 (2.15)	1.000	4.629	0.155 (0.34)	-	0.095 (0.35)
250	H	0.578 (1.76)	1.375	7.928	0.158 (0.28)	0.250 (0.004)	-
	L	0.566 (2.04)	1.029	15.722	0.157 (0.32)	-	0.088 (0.33)
	G	0.574 (2.07)	1.000	10.941	0.156 (0.30)	-	0.090 (0.35)
$N = 10000$							
100	H	0.587 (1.15)	1.285	2.899	0.156 (0.18)	0.250 (0.003)	-
	L	0.554 (1.44)	0.815	23.234	0.162 (2.24)	-	0.088 (0.242)
	G	0.587 (1.30)	1.000	3.253	0.156 (0.21)	-	0.093 (0.22)
250	H	0.582 (1.10)	1.884	4.603	0.157 (0.18)	0.250 (0.003)	-
	L	0.554 (1.14)	1.754	22.460	0.158 (0.22)	-	0.086 (0.23)
	G	0.570 (1.51)	1.000	11.583	0.156 (0.22)	-	0.089 (0.26)

H- HERRA, G - GCTA
