# Supplementary material for: Heritability Estimation using a Regularized Regression Approach (HERRA): Applicable to continuous, dichotomous or age-at-onset outcome
Source: PLoS One. 2017 Aug 16;12(8):e0181269. doi: 10.1371/journal.pone.0181269 (PMC5559077; doi:10.1371/journal.pone.0181269)
Supplement: S3 Table — Details of simulation results that are summarized by figures in the main text—continuous trait, one chromosome, Scenarios I and II. (PDF) [file pone.0181269.s003.pdf]

Heritability Estimation using a Regularized Regression Approach (HERRA): Applicable to Continuous, Dichotomous or Survival Outcome

Malka Gorfine[1,*], Sonja I Berndt[2], Jenny Chang-Claude[3], Michael Hoffmeister[4], Loic Le Marchand[5], John Potter[6], Martha L Slattery[7], Nir Keret[1], Ulrike Peters[6], Li Hsu[6,*]

1 Department of Statistics and Operation Research, Tel Aviv University, Tel Aviv, Israel

2 Division of Cancer Epidemiology and Genetics, National Cancer Institute, National Institutes of Health

3 Division of Cancer Epidemiology, German Cancer Research Center, Heidelberg, Germany

4 Division of Clinical Epidemiology and Aging Research, German Cancer Research Center, Heidelberg, Germany

5 Epidemiology Program, University of Hawaii Cancer Center

6 Public Health Sciences Division, Fred Hutchinson Cancer Research Center, Seattle, WA

7 Department of Internal Medicine, University of Utah Health Sciences Center

*** Correspondence: gorfinem@post.tau.ac.il, lih@fredhutch.org**

S3 Table: Tables of simulation results

The following S3 Table provides details of simulation results that are summarized by figures in the main text - continuous trait, one chromosome, Scenarios I and II.

Table S1. Simulation results of continuous trait, one chromosome, $N = 5000$, $p = 60$, $h^2 = 0.1$, $\sigma_e^2 = 1$, $\sigma_Y^2 = 1.111$, $\sigma_g^2 = 0.111$, all causal SNPs had a MAF $\leq \theta$: empirical mean (empirical SD$\times 10^2$), relative efficiency (RE), and mean-squared error (MSE)$\times 10^4$. For HERRA, RE is defined as the ratio of the variance of GCTA's estimator to the variance of HERRA's estimator. RE greater than 1 indicates that HERRA's estimator is more efficient.

	h^2			σ_e^2	σ_Y^2	σ_g^2
	mean (SD)	RE	MSE	mean (SD)	mean (SD)	mean (SD)
	Scenario I: All causal SNPs are with MAF $\leq \theta$					
	$\theta = 0.05$					
H	0.102 (1.18)	1.454	1.433	1.009 (1.96)	1.124 (2.08)	-
L	0.115 (1.31)	1.175	3.952	0.992(2.17)	-	0.129 (1.51)
G	0.104 (1.42)	1.000	2.198	1.008 (2.29)	-	0.117 (1.64)
	$\theta = 0.1$					
H	0.101 (1.33)	1.323	1.784	0.994 (2.12)	1.106 (2.01)	-
L	0.087 (1.45)	1.113	3.668	1.003 (2.33)	-	0.096 (1.63)
G	0.082 (1.53)	1.000	5.445	1.017 (2.47)	-	0.091 (1.70)
	$\theta = 0.5$					
H	0.103 (1.27)	1.310	1.713	0.997 (2.08)	1.112 (2.10)	-
L	0.100 (1.40)	1.077	1.953	0.999 (2.27)	-	0.111 (1.60)
G	0.102 (1.45)	1.000	2.147	0.997 (2.30)	-	0.113 (1.65)
	Scenario II: All causal SNPs are with MAF $\leq \theta$ and were excluded from the estimation procedure					
	$\theta = 0.05$					
H	0.104 (1.20)	1.400	1.634	1.006 (2.11)	1.123 (2.08)	-
L	0.092 (1.39)	1.044	2.605	1.020 (2.30)	-	0.103 (1.59)
G	0.076 (1.42)	1.000	7.669	1.039 (2.40)	-	0.086 (1.62)
	$\theta = 0.1$					
H	0.109 (1.31)	1.311	2.572	0.985 (2.22)	1.106 (2.01)	-
L	0.077 (1.42)	1.116	7.507	1.015 (2.35)	-	0.084 (1.59)
G	0.065 (1.50)	1.000	14.127	1.035 (2.49)	-	0.073 (1.66)
	$\theta = 0.5$					
H	0.104 (1.32)	1.141	1.945	0.996 (2.06)	1.112 (2.10)	-
L	0.093 (1.38)	1.044	2.322	1.007 (2.27)	-	0.104 (1.57)
G	0.090 (1.41)	1.000	2.536	1.008 (2.31)	-	0.103 (1.61)

H - HERRA, L - LDAK, G - GCTA
