# Supplementary material for: Heritability Estimation using a Regularized Regression Approach (HERRA): Applicable to continuous, dichotomous or age-at-onset outcome
Source: PLoS One. 2017 Aug 16;12(8):e0181269. doi: 10.1371/journal.pone.0181269 (PMC5559077; doi:10.1371/journal.pone.0181269)
Supplement: S4 Table — Details of simulation results that are summarized by figures in the main text—five chromosomes. (PDF) [file pone.0181269.s004.pdf]

Heritability Estimation using a Regularized Regression Approach (HERRA): Applicable to Continuous, Dichotomous or Survival Outcome

Malka Gorfine[1,*], Sonja I Berndt[2], Jenny Chang-Claude[3], Michael Hoffmeister[4], Loic Le Marchand[5], John Potter[6], Martha L Slattery[7], Nir Keret[1], Ulrike Peters[6], Li Hsu[6,*]

1 Department of Statistics and Operation Research, Tel Aviv University, Tel Aviv, Israel

2 Division of Cancer Epidemiology and Genetics, National Cancer Institute, National Institutes of Health

3 Division of Cancer Epidemiology, German Cancer Research Center, Heidelberg, Germany

4 Division of Clinical Epidemiology and Aging Research, German Cancer Research Center, Heidelberg, Germany

5 Epidemiology Program, University of Hawaii Cancer Center

6 Public Health Sciences Division, Fred Hutchinson Cancer Research Center, Seattle, WA

7 Department of Internal Medicine, University of Utah Health Sciences Center

*** Correspondence: gorfinem@post.tau.ac.il, lih@fredhutch.org**

S4 Table: Tables of simulation results

The following S4 Table provides details of simulation results that are summarized by figures in the main text - five chromosomes.

Table S1. Simulation results of five chromosomes, $M = 35,760$, $N = 10,000$, $p = 250$, all causal SNPs had a MAF $\leq \theta$: empirical mean (empirical SD$\times 10^2$), relative efficiency (RE), and mean-squared error (MSE)$\times 10^4$. For HERRA, RE is defined as the ratio of the variance of GCTA's estimator to the variance of HERRA's estimator. RE greater than 1 indicates that HERRA's estimator is more efficient.

	heritability			error variance	total variance	genetic variance
	mean (SD)	RE	MSE	mean (SD)	mean (SD)	mean (SD)
Continuous trait, true values: $h^2 = 0.1$; $\sigma_e^2 = 1$; $\sigma_Y^2 = 1.111$; $\sigma_g^2 = 0.111$						
$\theta = 0.5$						
H	0.104 (0.89)	1.525	0.977	0.995 (1.72)	1.110 (1.41)	-
L	0.098 (1.23)	0.800	1.545	1.001 (1.95)	-	0.109 (0.01)
G	0.099 (1.10)	1.000	1.231	1.000 (1.84)	-	0.109 (1.22)
Continuous trait, true values: $h^2 = 0.6$; $\sigma_e^2 = 1$; $\sigma_Y^2 = 2.5$; $\sigma_g^2 = 1.5$						
H	0.597 (0.72)	2.363	0.582	1.004 (1.84)	2.495 (3.14)	-
L	0.595 (1.01)	1.000	1.312	1.012 (2.40)	-	1.485 (0.03)
G	0.598 (1.01)	1.000	1.055	1.006 (2.36)	-	1.497 (3.57)
Dichotomous trait, true values: $h_l^2 = 0.1$; $\sigma_\epsilon^2 = 0.2341$; $\sigma_D^2 = 0.25$; $\sigma_{og}^2 = 0.0159$						
H	0.095 (1.12)	2.164	1.501	0.229 (0.18)	0.250 (0.004)	-
L	0.099 (1.85)	0.795	3.430	0.234 (0.29)	-	0.016 (0.29)
G	0.099 (1.65)	1.000	2.743	0.234 (0.27)	-	0.016 (0.27)
Dichotomous trait, true values: $h_l^2 = 0.6$; $\sigma_\epsilon^2 = 0.1545$; $\sigma_D^2 = 0.25$; $\sigma_{og}^2 = 0.0955$						
H	0.606 (1.18)	3.245	1.784	0.154 (0.19)	0.250 (0.003)	-
L	0.595 (1.88)	1.272	3.771	0.155 (0.30)	-	0.095 (0.31)
G	0.598 (2.12)	1.000	4.528	0.155 (0.28)	-	0.095 (0.30)
Continuous trait, Scenario II - all causal SNPs were excluded from the estimation procedure, true values: $h^2 = 0.1$; $\sigma_e^2 = 1$; $\sigma_Y^2 = 1.111$; $\sigma_g^2 = 0.111$						
$\theta = 0.05$						
H	0.094 (0.96)	1.273	1.344	1.003 (1.72)	1.106 (1.50)	-
L	0.069 (1.14)	0.898	10.769	1.029 (1.54)	-	0.077 (1.30)
G	0.057 (1.08)	1.000	18.088	1.043 (1.57)	-	0.064 (1.23)
$\theta = 0.1$						
H	0.095 (0.90)	1.033	1.050	1.002 (1.44)	1.107 (1.45)	-
L	0.074 (0.97)	0.880	7.742	1.025 (1.58)	-	0.082 (1.09)
G	0.062 (0.91)	1.000	14.153	1.038 (1.61)	-	0.069 (1.03)
$\theta = 0.5$						
H	0.106 (0.97)	1.128	1.326	0.992 (1.65)	1.109 (1.58)	-
L	0.088 (1.22)	1.230	2.947	1.013 (1.96)	-	0.097 (1.36)
G	0.090 (1.10)	1.000	1.011	1.011 (1.85)	-	0.099 (1.22)

H - HERRA, G - GCTA
