# Supplementary material for: Heritability Estimation using a Regularized Regression Approach (HERRA): Applicable to continuous, dichotomous or age-at-onset outcome
Source: PLoS One. 2017 Aug 16;12(8):e0181269. doi: 10.1371/journal.pone.0181269 (PMC5559077; doi:10.1371/journal.pone.0181269)
Supplement: S1 Text — A proof of consistency of HERRA estimator for continuous outcome is sketched, along with the asymptotic distribution of the estimator. (PDF) [file pone.0181269.s005.pdf]

Heritability Estimation using a Regularized Regression Approach (HERRA): Applicable to Continuous, Dichotomous or Survival Outcome

Malka Gorfine[1,*], Sonja I Berndt[2], Jenny Chang-Claude[3], Michael Hoffmeister[4], Loic Le Marchand[5], John Potter[6], Martha L Slattery[7], Nir Keret[1], Ulrike Peters[6], Li Hsu[6,*]

1 Department of Statistics and Operation Research, Tel Aviv University, Tel Aviv, Israel

2 Division of Cancer Epidemiology and Genetics, National Cancer Institute, National Institutes of Health

3 Division of Cancer Epidemiology, German Cancer Research Center, Heidelberg, Germany

4 Division of Clinical Epidemiology and Aging Research, German Cancer Research Center, Heidelberg, Germany

5 Epidemiology Program, University of Hawaii Cancer Center

6 Public Health Sciences Division, Fred Hutchinson Cancer Research Center, Seattle, WA

7 Department of Internal Medicine, University of Utah Health Sciences Center

*** Correspondence: gorfinem@post.tau.ac.il, lih@fredhutch.org**

S5 Text: Consistency for continuous outcome

Our method for estimating the error variance consists of an extra step, comparing to Fan et al. (2012). In this extra step, a variable-selection procedure is applied to the entire dataset, such as SIS or ITRRS. Consider first, a modified heritability estimator in which this extra step is excluded. From Theorem 2 of Fan et al. (2012) it follows that (given the regularity conditions 1 and 2 in Appendix A of Fan et al. 2012 and) as N goes to infinity when $p > N$ and given that causal loci are in perfect LD with genotyped SNPs, the limiting distribution of $\sqrt{N}(\widehat{\sigma}_e^2 - \sigma_e^2)$ is normal with mean 0 and variance $2\sigma_e^2$. Therefore, it is easy to show that as N goes to infinity, $\sqrt{N}\{(\widehat{\sigma}_Y^2, \widehat{\sigma}_e^2)^T - (\sigma_Y^2, \sigma_e^2)^T\}$ converges in distribution to a bivariate normally distributed random variable with zero mean and a covariance matrix Σ of dimension 2×2, where its components are $\Sigma_{11} = 2\sigma_Y^4$, $\Sigma_{22} = 2\sigma_e^4$, and $\Sigma_{12} = 2\sigma_e^4$. Hence, by the delta method, we conclude that the limiting distribution of $\sqrt{N}(\widehat{h}^2 - h^2)$ is also mean-zero normal with variance $4h^2(1-h^2)^2$.

With respect to the extra step, the SIS or ITRRS procedure, which we use in the filtering step, Fan et al. (2008, Theorem 1-3) showed that the procedure has a very high probability producing a model that contains the true model, and the size of the model is lower than the original dimension of predictors, as $N \to \infty$ when $p > N$ under some regularity conditions. Hence, the consistency of our heritability estimator holds as n goes to infinity. In practice, this extra step is to weed out variants that are unlikely to be associated with the phenotype rather identifying the model that best fits the data. Hence, the concern of over-fitting is less, comparing to Step 2 and 3, where we propose to split the dataset into training and estimation in order avoid over-fitting, following Fan et al. (2012).

References

1. Fan, J., Guo, S., and Hao, N. (2012) Variance estimation using refitted cross-validation in ultrahigh dimensional regression. *Journal of the Royal Statistical Society: Series B*, **74**, 37–65.

2. Brenner ,H., Chang-Claude ,J., Seiler ,C.M., Rickert ,A. and Hoffmeister ,M.

(2011) Protection from colorectal cancer after colonoscopy: a population-based, case-control study. *Ann. Intern. Med.*, **154**, 22–30.

3. Lilla C, Verla-Tebit E, Risch A, et al. (2006) Effect of NAT1 and NAT2 genetic polymorphisms on colorectal cancer risk associated with exposure to tobacco smoke and meat consumption. *Cancer Epidemiol Biomarkers Prev*, **15**, 99–107.

4. Slattery ,M.L., Potter ,J., Caan ,B., Edwards ,S., Coates ,A., Ma ,K.N. and Berry ,T.D. (1997) Energy balance and colon cancer–beyond physical activity. *Cancer Res.*, **57**, 75–80.

5. Le Marchand L, Hankin JH, Wilkens LR, Pierce LM, Franke A, Kolonel LN, Seifried A, Custer LJ, Chang W, Lum-Jones A, Donlon T (2001) Combined effects of well-done red meat, smoking, and rapid N-acetyltransferase 2 and CYP1A2 phenotypes in increasing colorectal cancer risk. *Cancer Epidemiol Biomarkers Prev,* **10**, 1259–1266.

6. Kolonel LN, Henderson BE, Hankin JH, Nomura AM, Wilkens LR, Pike MC, Stram DO, Monroe KR, Earle ME, Nagamine FS (2000) A multiethnic cohort in Hawaii and Los Angeles: baseline characteristics. *Am J Epidemiol*, **151**, 346–357.

7. Gohagan JK, Prorok PC, Hayes RB, Kramer BS (2000) The Prostate, Lung, Colorectal and Ovarian (PLCO) Cancer Screening Trial of the National Cancer Institute: history, organization, and status. Control Clin Trials 21:251S-272S

8. Prorok ,P.C., Andriole ,G.L., Bresalier ,R.S., Buys ,S.S., Chia ,D., Crawford ,E.D., Fogel ,R., Gelmann ,E.P., Gilbert ,F., Hasson ,M.A., et al. (2000) Design of the Prostate, Lung, Colorectal and Ovarian (PLCO) Cancer Screening Trial. *Control. Clin. Trials*, **21**, 273S–309S.

9. National Cancer Institute, (2009) Cancer Genetic Markers of Susceptibility (CGEMS) data website.

10. Yeager M, Chatterjee N, Ciampa J, Jacobs KB, Gonzalez-Bosquet J, Hayes RB, Kraft P, Wacholder S, Orr N, Berndt S, Yu K, Hutchinson A, Wang Z, Amundadottir L, Feigelson HS, Thun MJ, Diver WR, Albanes D, Virtamo J, Weinstein S, Schumacher FR, Cancel-Tassin G, Cussenot O, Valeri A, Andriole

GL, Crawford ED, Haiman CA, Henderson B, Kolonel L, Le ML, Siddiq A, Riboli E, Key TJ, Kaaks R, Isaacs W, Isaacs S, Wiley KE, Gronberg H, Wiklund F, Stattin P, Xu J, Zheng SL, Sun J, Vatten LJ, Hveem K, Kumle M, Tucker M, Gerhard DS, Hoover RN, Fraumeni JF, Jr., Hunter DJ, Thomas G, Chanock SJ (2009) Identification of a new prostate cancer susceptibility locus on chromosome 8q24. *Nature genetics*, **41**, 1055–1057.

11. Landi MT, Chatterjee N, Yu K, Goldin LR, Goldstein AM, Rotunno M, Mirabello L, Jacobs K, Wheeler W, Yeager M, Bergen AW, Li Q, Consonni D, Pesatori AC, Wacholder S, Thun M, Diver R, Oken M, Virtamo J, Albanes D, Wang Z, Burdette L, Doheny KF, Pugh EW, Laurie C, Brennan P, Hung R, Gaborieau V, McKay JD, Lathrop M, McLaughlin J, Wang Y, Tsao MS, Spitz MR, Wang Y, Krokan H, Vatten L, Skorpen F, Arnesen E, Benhamou S, Bouchard C, Metsapalu A, Vooder T, Nelis M, Valk K, Field JK, Chen C, Goodman G, Sulem P, Thorleifsson G, Rafnar T, Eisen T, Sauter W, Rosenberger A, Bickeboller H, Risch A, Chang-Claude J, Wichmann HE, Stefansson K, Houlston R, Amos CI, Fraumeni JF, Jr., Savage SA, Bertazzi PA, Tucker MA, Chanock S, Caporaso NE (2009) A genome-wide association study of lung cancer identifies a region of chromosome 5p15 associated with risk for adenocarcinoma. *Am J Hum Genet*, **85**, 679–691.

12. White E, Patterson RE, Kristal AR, Thornquist M, King I, Shattuck AL, Evans I, Satia-Abouta J, Littman AJ, Potter JD (2004) VITamins And Lifestyle cohort study: study design and characteristics of supplement users. *Am J Epidemiol*, **159**,83–93.

13. Hays J, Hunt JR, Hubbell FA, Anderson GL, Limacher M, Allen C, Rossouw JE (2003) The Women's Health Initiative recruitment methods and results. *Ann Epidemiol*, **13**, S18-S77.

14. The Women's Health Initiative Study Group (1998) Design of the Women's Health Initiative clinical trial and observational study. *Control Clin Trials*, **19**, 61–109.

15. Bergstralh, Kosanke JL (1995) *Computerized matching of cases to controls*, 56 edn Department of Health Sciences Research, Mayo Clinic, Rochester MN.
