# Supplementary material for: Heritability Estimation using a Regularized Regression Approach (HERRA): Applicable to continuous, dichotomous or age-at-onset outcome
Source: PLoS One. 2017 Aug 16;12(8):e0181269. doi: 10.1371/journal.pone.0181269 (PMC5559077; doi:10.1371/journal.pone.0181269)
Supplement: S3 Text — The weighted bootstrap variance estimator is described in details. (PDF) [file pone.0181269.s007.pdf]

Heritability Estimation using a Regularized Regression Approach (HERRA): Applicable to Continuous, Dichotomous or Survival Outcome

Malka Gorfine[1,*], Sonja I Berndt[2], Jenny Chang-Claude[3], Michael Hoffmeister[4], Loic Le Marchand[5], John Potter[6], Martha L Slattery[7], Nir Keret[1], Ulrike Peters[6], Li Hsu[6,*]

1 Department of Statistics and Operation Research, Tel Aviv University, Tel Aviv, Israel

2 Division of Cancer Epidemiology and Genetics, National Cancer Institute, National Institutes of Health

3 Division of Cancer Epidemiology, German Cancer Research Center, Heidelberg, Germany

4 Division of Clinical Epidemiology and Aging Research, German Cancer Research Center, Heidelberg, Germany

5 Epidemiology Program, University of Hawaii Cancer Center

6 Public Health Sciences Division, Fred Hutchinson Cancer Research Center, Seattle, WA

7 Department of Internal Medicine, University of Utah Health Sciences Center

*** Correspondence: gorfinem@post.tau.ac.il, lih@fredhutch.org**

S7 Text: Weighted-bootstrap variance estimator

For the weighted bootstrap, a sample of independent identically distributed weights from unit exponential distribution is drawn B times, $\omega_1^{(b)}, \ldots, \omega_N^{(b)}$, $b = 1, \ldots, B$. The bth bootstrap dataset consists of the original dataset and the bth sequence of weights. For each bootstrap sample, apply Step 2 – Step 4 with weighted lasso and weighted ordinary least squares method, and get $\widehat{\sigma}_e^{2(b)}$, $b = 1, \ldots, B$. Let $\widehat{\sigma}_Y^{2(b)} = \sum_{i=1}^N \omega_i^{(b)}(Y_i - \bar{Y}_\omega^{(b)})^2/(\nu_1^{(b)} - \nu_2^{(b)}/\nu_1^{(b)})$ where $\bar{Y}_\omega^{(b)} = \sum_{i=1}^N \omega_i^{(b)} Y_i/\nu_1^{(b)}$, $\nu_1^{(b)} = \sum_{i=1}^N \omega_i^{(b)}$ and $\nu_2^{(b)} = \sum_{i=1}^N \omega_i^{(b)2}$, $b = 1, \ldots, B$. Then, the variance estimator and confidence interval are based on the series $\widehat{h}^{2(b)} = 1 - \widehat{\sigma}_e^{2(b)}/\widehat{\sigma}_Y^{2(b)}$, $b = 1 \ldots, B$. Our simulation results show that this weighted bootstrap approach with lasso's fixed complexity parameter of lasso works reasonably well in terms of estimating the standard error and empirical coverage rate, whereas in some cases it tends to overestimate the true variance of $\widehat{h}^2$. For example, with one chromosome of $M = 9344$ SNPs, $N = 10,000$ observations, $p = 250$ causal SNPs, $h^2 = 0.6$ and 0/1 outcome, the mean of the estimated heritability on liability scale was 0.582, the empirical standard error was 0.0114, the mean of the weighted bootstrap estimated heritability on liability scale was 0.607, and the weighted bootstrap standard error was 0.0158. Then, the 95% coverage rate was 100.

References

1. Fan, J., Guo, S., and Hao, N. (2012) Variance estimation using refitted cross-validation in ultrahigh dimensional regression. *Journal of the Royal Statistical Society: Series B*, **74**, 37–65.

2. Brenner ,H., Chang-Claude ,J., Seiler ,C.M., Rickert ,A. and Hoffmeister ,M. (2011) Protection from colorectal cancer after colonoscopy: a population-based, case-control study. *Ann. Intern. Med.*, **154**, 22–30.

3. Lilla C, Verla-Tebit E, Risch A, et al. (2006) Effect of NAT1 and NAT2 genetic polymorphisms on colorectal cancer risk associated with exposure to tobacco smoke and meat consumption. *Cancer Epidemiol Biomarkers Prev*, **15**, 99–107.

4. Slattery ,M.L., Potter ,J., Caan ,B., Edwards ,S., Coates ,A., Ma ,K.N. and Berry ,T.D. (1997) Energy balance and colon cancer–beyond physical activity. *Cancer Res.*, **57**, 75–80.

5. Le Marchand L, Hankin JH, Wilkens LR, Pierce LM, Franke A, Kolonel LN, Seifried A, Custer LJ, Chang W, Lum-Jones A, Donlon T (2001) Combined effects of well-done red meat, smoking, and rapid N-acetyltransferase 2 and CYP1A2 phenotypes in increasing colorectal cancer risk. *Cancer Epidemiol Biomarkers Prev,* **10**, 1259–1266.

6. Kolonel LN, Henderson BE, Hankin JH, Nomura AM, Wilkens LR, Pike MC, Stram DO, Monroe KR, Earle ME, Nagamine FS (2000) A multiethnic cohort in Hawaii and Los Angeles: baseline characteristics. *Am J Epidemiol*, **151**, 346–357.

7. Gohagan JK, Prorok PC, Hayes RB, Kramer BS (2000) The Prostate, Lung, Colorectal and Ovarian (PLCO) Cancer Screening Trial of the National Cancer Institute: history, organization, and status. Control Clin Trials 21:251S-272S

8. Prorok ,P.C., Andriole ,G.L., Bresalier ,R.S., Buys ,S.S., Chia ,D., Crawford ,E.D., Fogel ,R., Gelmann ,E.P., Gilbert ,F., Hasson ,M.A., et al. (2000) Design of the Prostate, Lung, Colorectal and Ovarian (PLCO) Cancer Screening Trial. *Control. Clin. Trials*, **21**, 273S–309S.

9. National Cancer Institute, (2009) Cancer Genetic Markers of Susceptibility (CGEMS) data website.

10. Yeager M, Chatterjee N, Ciampa J, Jacobs KB, Gonzalez-Bosquet J, Hayes RB, Kraft P, Wacholder S, Orr N, Berndt S, Yu K, Hutchinson A, Wang Z, Amundadottir L, Feigelson HS, Thun MJ, Diver WR, Albanes D, Virtamo J, Weinstein S, Schumacher FR, Cancel-Tassin G, Cussenot O, Valeri A, Andriole GL, Crawford ED, Haiman CA, Henderson B, Kolonel L, Le ML, Siddiq A, Riboli E, Key TJ, Kaaks R, Isaacs W, Isaacs S, Wiley KE, Gronberg H, Wiklund F, Stattin P, Xu J, Zheng SL, Sun J, Vatten LJ, Hveem K, Kumle M, Tucker M, Gerhard DS, Hoover RN, Fraumeni JF, Jr., Hunter DJ, Thomas G, Chanock SJ (2009) Identification of a new prostate cancer susceptibility locus on chromosome 8q24. *Nature genetics*, **41**, 1055–1057.

11. Landi MT, Chatterjee N, Yu K, Goldin LR, Goldstein AM, Rotunno M, Mirabello L, Jacobs K, Wheeler W, Yeager M, Bergen AW, Li Q, Consonni D, Pesatori AC, Wacholder S, Thun M, Diver R, Oken M, Virtamo J, Albanes D, Wang Z, Burdette L, Doheny KF, Pugh EW, Laurie C, Brennan P, Hung R, Gaborieau V, McKay JD, Lathrop M, McLaughlin J, Wang Y, Tsao MS, Spitz MR, Wang Y, Krokan H, Vatten L, Skorpen F, Arnesen E, Benhamou S, Bouchard C, Metsapalu A, Vooder T, Nelis M, Valk K, Field JK, Chen C, Goodman G, Sulem P, Thorleifsson G, Rafnar T, Eisen T, Sauter W, Rosenberger A, Bickeboller H, Risch A, Chang-Claude J, Wichmann HE, Stefansson K, Houlston R, Amos CI, Fraumeni JF, Jr., Savage SA, Bertazzi PA, Tucker MA, Chanock S, Caporaso NE (2009) A genome-wide association study of lung cancer identifies a region of chromosome 5p15 associated with risk for adenocarcinoma. *Am J Hum Genet*, **85**, 679–691.

12. White E, Patterson RE, Kristal AR, Thornquist M, King I, Shattuck AL, Evans I, Satia-Abouta J, Littman AJ, Potter JD (2004) VITamins And Lifestyle cohort study: study design and characteristics of supplement users. *Am J Epidemiol*, **159**,83–93.

13. Hays J, Hunt JR, Hubbell FA, Anderson GL, Limacher M, Allen C, Rossouw JE (2003) The Women's Health Initiative recruitment methods and results. *Ann Epidemiol*, **13**, S18-S77.

14. The Women's Health Initiative Study Group (1998) Design of the Women's Health Initiative clinical trial and observational study. *Control Clin Trials*, **19**, 61–109.

15. Bergstralh, Kosanke JL (1995) *Computerized matching of cases to controls*, 56 edn Department of Health Sciences Research, Mayo Clinic, Rochester MN.
