# Supplementary material for: Heritability Estimation using a Regularized Regression Approach (HERRA): Applicable to continuous, dichotomous or age-at-onset outcome
Source: PLoS One. 2017 Aug 16;12(8):e0181269. doi: 10.1371/journal.pone.0181269 (PMC5559077; doi:10.1371/journal.pone.0181269)
Supplement: S4 Text — A Description of the study populations included in Genome-wide Association Study Analysis (GWAS) is detailed. (PDF) [file pone.0181269.s008.pdf]

Heritability Estimation using a Regularized Regression Approach (HERRA): Applicable to Continuous, Dichotomous or Survival Outcome

Malka Gorfine[1,*], Sonja I Berndt[2], Jenny Chang-Claude[3], Michael Hoffmeister[4], Loic Le Marchand[5], John Potter[6], Martha L Slattery[7], Nir Keret[1], Ulrike Peters[6], Li Hsu[6,*]

1 Department of Statistics and Operation Research, Tel Aviv University, Tel Aviv, Israel

2 Division of Cancer Epidemiology and Genetics, National Cancer Institute, National Institutes of Health

3 Division of Cancer Epidemiology, German Cancer Research Center, Heidelberg, Germany

4 Division of Clinical Epidemiology and Aging Research, German Cancer Research Center, Heidelberg, Germany

5 Epidemiology Program, University of Hawaii Cancer Center

6 Public Health Sciences Division, Fred Hutchinson Cancer Research Center, Seattle, WA

7 Department of Internal Medicine, University of Utah Health Sciences Center

*** Correspondence: gorfinem@post.tau.ac.il, lih@fredhutch.org**

S8 Text: Description of study populations included in Genome-wide Association Study Analysis (GWAS)

Darmkrebs: Chancen der Verhütung durch Screening (DACHS) [2, 3]. This German study was initiated as a large population-based case-control study in 2003 in the Rhine-Neckar-Odenwald region (southwest region of Germany) to assess the potential of endoscopic screening for reduction of colorectal cancer risk and to investigate etiologic determinants of the disease, particularly lifestyle/environmental factors and genetic factors. Cases with a first diagnosis of invasive colorectal cancer (ICD-10 codes C18-C20) who were at least 30 years of age (no upper age limit), German speaking, a resident in the study region, and mentally and physically able to participate in a one-hour interview, were recruited by their treating physicians either in the hospital a few days after surgery, or by mail after discharge from the hospital. Cases were confirmed based on histologic reports and hospital discharge letters following diagnosis of colorectal cancer. All hospitals treating colorectal cancer patients in the study region participated. Based on estimates from population-based cancer registries, more than 50% of all potentially eligible patients with incident colorectal cancer in the study region were included. Community-based controls were randomly selected from population registries, employing frequency matching with respect to age (5-year groups), sex, and county of residence. Controls with a history of colorectal cancer were excluded. Controls were contacted by mail and follow-up calls. The participation rate was 51%. During an in-person interview, data were collected on demographics, medical history, family history of CRC, and various life-style factors, as were blood and mouthwash samples. The Set 1 scan consisted of a subset of participants recruited up to 2007, and samples were frequency-matched on age and sex. The Set 2 scan consisted of additional subjects that were recruited up to 2010 as part of this ongoing study.

Diet, Activity, and Lifestyle Study (DALS) [4]. DALS is a population-based case-control study of colon cancer. Participants were recruited between 1991 and 1994 from three locations: the Kaiser Permanente Medical Care Program (KPMCP) of Northern California, an eight-county area in Utah, and the metropolitan Twin Cities area of Minnesota. Eligibility criteria for cases included age at diagnosis between 30 and

79 years, diagnosis with first primary colon cancer (ICD-O-2 codes 18.0 and 18.2-18.9) between October 1st 1991 and September 30th 1994, ability to speak English, and competency to complete the interview. Individuals with cancer of the rectosigmoid junction or rectum were excluded, as were those with a pathology report noting familial adenomatous polyposis, Crohn's disease, or ulcerative colitis. A rapid-reporting system was used to identify all incident cases of colon cancer resulting in the majority of cases being interviewed within four months of diagnosis. Controls from KPMCP were randomly selected from membership lists. In Utah, controls under 65 years of age were randomly selected through random-digit dialing and driver license lists. Controls, 65 years of age and older, were randomly selected from Health Care Financing Administration lists. In Minnesota, controls were identified from Minnesota driver's license or state ID lists. Cases and controls were matched to cases by 5-year age groups and sex. The Set I scan consisted of a subset of the study designed above, from Utah, Minnesota, and KPMCP, and was restricted to subjects who self-reported as White non-Hispanic. The Set 2 scan consisted of subjects from Utah and Minnesota that were not genotyped in Set 1. Set 2 was restricted to subjects who self-reported as White non-Hispanic and those that had appropriate consent to post data to dbGaP.

Hawai'i Colorectal Cancer Studies 2 & 3 (Colo2&3) [5]. Patients with colorectal cancer were identified through the rapid reporting system of the Hawaii SEER registry and consisted of all Japanese, Caucasian, and Native Hawaiian residents of Oahu who were newly diagnosed with an adenocarcinoma of the colon or rectum between January 1994 and August 1998. Control subjects were selected from participants in an on-going population-based health survey conducted by the Hawaii State Department of Health and from Health Care Financing Administration participants. Controls were matched to cases by sex, ethnicity, and age (within two years). Personal interviews were obtained from 768 matched pairs, resulting in a participation rate of 58.2% for cases and 53.2% for controls. A questionnaire, administered during an in-person interview, included questions about demographics, lifetime history of tobacco, alcohol use, aspirin use, physical activity, personal medical history, family history of colorectal cancer, height and weight, diet (Food Frequency Questionnaire), and postmenopausal hormone use. A blood sample was obtained from

548 (71%) of interviewed cases and 662 (86%) of interviewed controls. SEER staging information was extracted from the Hawaii Tumor Registry. In GECCO, self-reported Caucasian subjects with DNA, clinical and epidemiologic data were selected for genotyping.

Multiethnic Cohort Study (MEC) [6]. MEC was initiated in 1993 to investigate the impact of dietary and environmental factors on major chronic diseases, particularly cancer, in ethnically diverse populations in Hawaii and California. The study recruited 96,810 men and 118,441 women aged 45 to 75 years between 1993 and 1996. Incident colorectal cancer cases occurring since January 1995 and controls were contacted for blood or saliva samples. The median interval between diagnosis and blood draw was 14 months (interquartile range, 10-19mo) among cases and the participation rate 74%. A sample of cohort participants was randomly selected to serve as controls at the onset of the nested case-control study (participation rate 66%). The selection was stratified by sex, age, and race/ethnicity. Colorectal cancer cases are identified through the Rapid Reporting System of the Hawai'i Tumor Registry and through quarterly linkage to the Los Angeles County Cancer Surveillance Program. Both registries are members of SEER. In GECCO, self-reported White subjects from the nested case-control study described above with DNA, and clinical and epidemiologic data were selected for genotyping.

Prostate, Lung, Colorectal, and Ovarian Cancer Screening Trial (PLCO). PLCO enrolled 154,934 participants (men and women, aged between 55 and 74 years) at ten centers into a large, randomized, two-arm trial to determine the effectiveness of screening to reduce cancer mortality. Sequential blood samples were collected from participants assigned to the screening arm. Participation was 93% at the baseline blood draw. In the observational (control) arm, buccal cells were collected via mail using the "swish-and-spit" protocol and participation rate was 65%. Details of this study have been previously described [7, 8] and are available online (http://dcp.cancer.gov/plco).

The Set 1 scan included a subset of 577 colon cancer cases self-reported as being non-Hispanic White with available DNA samples, questionnaire data, and appropriate consent for ancillary epidemiologic studies. Cases were excluded if they had a history of inflammatory bowel disease, polyps, polyposis syndrome or cancer (excluding basal or

squamous cell skin cancer). Controls came from the Cancer Genetic Markers of Susceptibility (CGEMS) prostate cancer scan [9, 10] (all male) and the GWAS of Lung Cancer and Smoking [11] (enriched for smokers) along with an additional 92 non-Hispanic White female controls. For the Set 2 scan, cases were colorectal cancers from both arms of the trial, which were not already included in Set 1. Samples were excluded if participants did not sign appropriate consents, if DNA was unavailable, if baseline questionnaire data with follow-up were unavailable, if they had a history of colon cancer prior to the trial, if they had a rare cancer, and if they were already in a colon GWAS, or if they were a control in the prostate or lung populations. Controls were frequency matched 1:1 to cases without replacement, and cases were not eligible to be controls. Matching criteria were age at enrollment (two year blocks), enrollment date (two year blocks), sex, race / ethnicity, trial arm, and study year of diagnosis (i.e. controls must have been cancer-free into the case's year of diagnosis).

VITamins And Lifestyle (VITAL). The VITamins And Lifestyle (VITAL) cohort comprises of 77,721 Washington State men and women aged 50 to 76 years, recruited from 2000 to 2002 to investigate the association of supplement use and lifestyle factors with cancer risk. Subjects were recruited by mail, from October 2000 to December 2002, using names purchased from a commercial mailing list. All subjects competed a 24-page questionnaire and buccal-cell specimens for DNA was self-collected by 70% of the participants. Subjects are followed for cancer by linkage to the western Washington SEER cancer registry and are censored when they move out of the area covered by the registry, or at time of death. Details of this study have been previously described [12]. In GECCO, a nested case-control set was genotyped. Samples included colorectal cancer cases with DNA, excluding subjects with colorectal cancer before baseline, in situ cases, (large cell) neuro-endocrine carcinoma, squamous cell carcinoma, carcinoid tumor, Goblet cell carcinoid, any type of lymphoma, including non-Hodgkin, Mantle cell, large B-cell, or follicular lymphoma. Controls were matched on age at enrollment (within one year), enrollment date (within one year), sex, and race / ethnicity. One control was randomly selected per case among all controls that matched on the four factors above and where the control follow-up time was greater than follow-up time of the case until diagnosis.

Women's Health Initiative (WHI). WHI is a long-term health study of 161,808 post-menopausal women aged 50 to 79 years at 40 clinical centers throughout the U.S. WHI comprises a Clinical Trial (CT) arm, an Observational Study (OS) arm, and several extension studies. The details of WHI have been previously described [13, 14] and are available online (https://cleo.whi. org/SitePages/Home.aspx). In GECCO, Set 1 cases were selected from the September 12, 2005 database and were comprised of centrally adjudicated colon cancer cases from the Observational Study (OS) who self-reported as White. Controls were first selected among controls previously genotyped as part of a Hip Fracture GWAS conducted within the WHI OS and matched to cases by age (within three years) enrollment date (within 365 days), hysterectomy status, and prevalent conditions at baseline. For 37 cases, there was no control match in the Hip Fracture GWAS. For these participants, we identified a matched control in the WHI OS based on the same criteria. In the Set 2 scan, cases were selected from the August 2009 database and were comprised of centrally adjudicated colon and colorectal cancer cases from the OS and CT who were not genotyped in Set 1. In addition, case and control participants were subject to the following exclusion criteria: a prior history of colorectal cancer at baseline, IRB approval not available for data submission into dbGaP, and insufficient DNA available. Matching criteria included age (within years), race/ethnicity, WHI date (within three years), WHI Calcium and Vitamin D study date (within three years), and randomization arms (OS flag, hormone therapy assignments, dietary modification assignments, calcium/vitamin D assignments). In addition, they were matched on the four regions of randomization centers. Each case was matched with one control (1:1) that exactly met the matching criteria. Control selection was done in a time-forward manner, selecting one control for each case first from the risk set at the time of the case event. The matching algorithm was allowed to select the closest match based on a criterion to minimize an overall distance measure [15]. Each matching factor was given the same weight. Additional available controls that were genotyped as part of the Hip Fracture GWAS were included to improve power.

References

1. Fan, J., Guo, S., and Hao, N. (2012) Variance estimation using refitted cross-validation in ultrahigh dimensional regression. *Journal of the Royal Statistical Society: Series B*, **74**, 37–65.

2. Brenner ,H., Chang-Claude ,J., Seiler ,C.M., Rickert ,A. and Hoffmeister ,M. (2011) Protection from colorectal cancer after colonoscopy: a population-based, case-control study. *Ann. Intern. Med.*, **154**, 22–30.

3. Lilla C, Verla-Tebit E, Risch A, et al. (2006) Effect of NAT1 and NAT2 genetic polymorphisms on colorectal cancer risk associated with exposure to tobacco smoke and meat consumption. *Cancer Epidemiol Biomarkers Prev*, **15**, 99–107.

4. Slattery ,M.L., Potter ,J., Caan ,B., Edwards ,S., Coates ,A., Ma ,K.N. and Berry ,T.D. (1997) Energy balance and colon cancer–beyond physical activity. *Cancer Res.*, **57**, 75–80.

5. Le Marchand L, Hankin JH, Wilkens LR, Pierce LM, Franke A, Kolonel LN, Seifried A, Custer LJ, Chang W, Lum-Jones A, Donlon T (2001) Combined effects of well-done red meat, smoking, and rapid N-acetyltransferase 2 and CYP1A2 phenotypes in increasing colorectal cancer risk. *Cancer Epidemiol Biomarkers Prev,* **10**, 1259–1266.

6. Kolonel LN, Henderson BE, Hankin JH, Nomura AM, Wilkens LR, Pike MC, Stram DO, Monroe KR, Earle ME, Nagamine FS (2000) A multiethnic cohort in Hawaii and Los Angeles: baseline characteristics. *Am J Epidemiol*, **151**, 346–357.

7. Gohagan JK, Prorok PC, Hayes RB, Kramer BS (2000) The Prostate, Lung, Colorectal and Ovarian (PLCO) Cancer Screening Trial of the National Cancer Institute: history, organization, and status. Control Clin Trials 21:251S-272S

8. Prorok ,P.C., Andriole ,G.L., Bresalier ,R.S., Buys ,S.S., Chia ,D., Crawford ,E.D., Fogel ,R., Gelmann ,E.P., Gilbert ,F., Hasson ,M.A., et al. (2000) Design of the Prostate, Lung, Colorectal and Ovarian (PLCO) Cancer Screening Trial. *Control. Clin. Trials*, **21**, 273S–309S.

9. National Cancer Institute, (2009) Cancer Genetic Markers of Susceptibility (CGEMS) data website.

10. Yeager M, Chatterjee N, Ciampa J, Jacobs KB, Gonzalez-Bosquet J, Hayes RB, Kraft P, Wacholder S, Orr N, Berndt S, Yu K, Hutchinson A, Wang Z, Amundadottir L, Feigelson HS, Thun MJ, Diver WR, Albanes D, Virtamo J, Weinstein S, Schumacher FR, Cancel-Tassin G, Cussenot O, Valeri A, Andriole GL, Crawford ED, Haiman CA, Henderson B, Kolonel L, Le ML, Siddiq A, Riboli E, Key TJ, Kaaks R, Isaacs W, Isaacs S, Wiley KE, Gronberg H, Wiklund F, Stattin P, Xu J, Zheng SL, Sun J, Vatten LJ, Hveem K, Kumle M, Tucker M, Gerhard DS, Hoover RN, Fraumeni JF, Jr., Hunter DJ, Thomas G, Chanock SJ (2009) Identification of a new prostate cancer susceptibility locus on chromosome 8q24. *Nature genetics*, **41**, 1055–1057.

11. Landi MT, Chatterjee N, Yu K, Goldin LR, Goldstein AM, Rotunno M, Mirabello L, Jacobs K, Wheeler W, Yeager M, Bergen AW, Li Q, Consonni D, Pesatori AC, Wacholder S, Thun M, Diver R, Oken M, Virtamo J, Albanes D, Wang Z, Burdette L, Doheny KF, Pugh EW, Laurie C, Brennan P, Hung R, Gaborieau V, McKay JD, Lathrop M, McLaughlin J, Wang Y, Tsao MS, Spitz MR, Wang Y, Krokan H, Vatten L, Skorpen F, Arnesen E, Benhamou S, Bouchard C, Metsapalu A, Vooder T, Nelis M, Valk K, Field JK, Chen C, Goodman G, Sulem P, Thorleifsson G, Rafnar T, Eisen T, Sauter W, Rosenberger A, Bickeboller H, Risch A, Chang-Claude J, Wichmann HE, Stefansson K, Houlston R, Amos CI, Fraumeni JF, Jr., Savage SA, Bertazzi PA, Tucker MA, Chanock S, Caporaso NE (2009) A genome-wide association study of lung cancer identifies a region of chromosome 5p15 associated with risk for adenocarcinoma. *Am J Hum Genet*, **85**, 679–691.

12. White E, Patterson RE, Kristal AR, Thornquist M, King I, Shattuck AL, Evans I, Satia-Abouta J, Littman AJ, Potter JD (2004) VITamins And Lifestyle cohort study: study design and characteristics of supplement users. *Am J Epidemiol*, **159**,83–93.

13. Hays J, Hunt JR, Hubbell FA, Anderson GL, Limacher M, Allen C, Rossouw JE (2003) The Women's Health Initiative recruitment methods and results. *Ann Epidemiol*, **13**, S18-S77.

14. The Women's Health Initiative Study Group (1998) Design of the Women's Health Initiative clinical trial and observational study. *Control Clin Trials*, **19**, 61–109.

15. Bergstralh, Kosanke JL (1995) *Computerized matching of cases to controls*, 56 edn Department of Health Sciences Research, Mayo Clinic, Rochester MN.
