# Supplementary material for: Heritability Estimation using a Regularized Regression Approach (HERRA): Applicable to continuous, dichotomous or age-at-onset outcome
Source: PLoS One. 2017 Aug 16;12(8):e0181269. doi: 10.1371/journal.pone.0181269 (PMC5559077; doi:10.1371/journal.pone.0181269)
Supplement: S5 Text — Details on quality assurance and quality control of the colorectal cancer GWAS dataset. (PDF) [file pone.0181269.s009.pdf]

Heritability Estimation using a Regularized Regression Approach (HERRA): Applicable to Continuous, Dichotomous or Survival Outcome

Malka Gorfine[1,*], Sonja I Berndt[2], Jenny Chang-Claude[3], Michael Hoffmeister[4], Loic Le Marchand[5], John Potter[6], Martha L Slattery[7], Nir Keret[1], Ulrike Peters[6], Li Hsu[6,*]

1 Department of Statistics and Operation Research, Tel Aviv University, Tel Aviv, Israel

2 Division of Cancer Epidemiology and Genetics, National Cancer Institute, National Institutes of Health

3 Division of Cancer Epidemiology, German Cancer Research Center, Heidelberg, Germany

4 Division of Clinical Epidemiology and Aging Research, German Cancer Research Center, Heidelberg, Germany

5 Epidemiology Program, University of Hawaii Cancer Center

6 Public Health Sciences Division, Fred Hutchinson Cancer Research Center, Seattle, WA

7 Department of Internal Medicine, University of Utah Health Sciences Center

*** Correspondence: gorfinem@post.tau.ac.il, lih@fredhutch.org**

S9 Text: Details on quality assurance and quality control of the colorectal cancer GWAS dataset

Table S1. Quality assurance and quality control

Study	Sample Exclusions[1] N	Duplicate Concordance %	Sample Call Rate Mean	SNP Exclusions[2] N	SNPs Passing QC N	SNP Call Rate Mean
Colo2&3	42	100.0%	99.90%	30,446	252,176	99.95%
DACHS1	227	99.90%	99.93%	33,588	252,208	99.90%
DALS2	5	100.0%	99.94%	32,885	250,320	99.94%
MEC	61	100.0%	99.97%	34,494	259,364	99.96%
PLCO2	31	99.90%	99.80%	38,655	253,702	99.90%
VITAL	163	99.90%	99.81%	36,805	243,625	99.89%
WHI2	42	100.0%	99.96%	30,446	251,707	99.96%

[1]Samples were excluded for duplicate errors, gender errors, a call rate $< 97\%$, outlying the CEU HapMap2 cluster in PCA, heterozygosity, and concordance with non-duplicates samples $> 65\%$.
[2]Directly genotyped SNPs were excluded for a call rate ¡ 98%, Hardy Weinberg Equilibrium (HWE) $< 10^{-4}$, and minor allele frequency (MAF$< 1\%$).
