# Supplementary material for: Heritability Estimation using a Regularized Regression Approach (HERRA): Applicable to continuous, dichotomous or age-at-onset outcome
Source: PLoS One. 2017 Aug 16;12(8):e0181269. doi: 10.1371/journal.pone.0181269 (PMC5559077; doi:10.1371/journal.pone.0181269)
Supplement: S6 Text — Effect sizes used in the simulations with p = 100 causal SNPs and h2 = 0.1. (PDF) [file pone.0181269.s010.pdf]

Heritability Estimation using a Regularized Regression Approach (HERRA): Applicable to Continuous, Dichotomous or Survival Outcome

Malka Gorfine[1,*], Sonja I Berndt[2], Jenny Chang-Claude[3], Michael Hoffmeister[4], Loic Le Marchand[5], John Potter[6], Martha L Slattery[7], Nir Keret[1], Ulrike Peters[6], Li Hsu[6,*]

1 Department of Statistics and Operation Research, Tel Aviv University, Tel Aviv, Israel

2 Division of Cancer Epidemiology and Genetics, National Cancer Institute, National Institutes of Health

3 Division of Cancer Epidemiology, German Cancer Research Center, Heidelberg, Germany

4 Division of Clinical Epidemiology and Aging Research, German Cancer Research Center, Heidelberg, Germany

5 Epidemiology Program, University of Hawaii Cancer Center

6 Public Health Sciences Division, Fred Hutchinson Cancer Research Center, Seattle, WA

7 Department of Internal Medicine, University of Utah Health Sciences Center

*** Correspondence: gorfinem@post.tau.ac.il, lih@fredhutch.org**

S10 Text: Effect sizes used in the simulations with $p = 100$ causal SNPs and $h^2 = 0.1$.

-0.0084 0.0359 0.0149 0.0260 0.0259 -0.0386 0.0542 0.0019 0.0203 0.0299 -0.0269 0.0056 -0.0007 -0.0880 0.0031 -0.0322 -0.0313 -0.0373 -0.0214 0.0348 -0.0031 -0.0177 -0.0158 -0.0371 0.0520 -0.0299 -0.0743 -0.0327 0.0582 -0.0409 0.0024 0.0094 0.0107 0.0010 0.0289 -0.0056 0.0044 0.0336 -0.0348 0.0163 0.0417 -0.0165 -0.0021 0.0226 0.0168 -0.0461 0.0657 -0.0182 0.0117 0.0201 -0.0632 0.0213 0.0120 0.0048 -0.0543 0.0081 -0.0832 -0.0366 0.0143 -0.0224 -0.0244 -0.0126 0.0691 -0.0046 -0.0269 -0.0246 -0.0287 0.0459 -0.0178 0.0064 -0.0119 -0.0159 -0.0323 -0.0424 0.0069 0.0354 0.0117 0.1298 -0.0041 0.0081 0.0433 -0.0059 0.0171 -0.0149 0.0058 -0.0002 0.0284 -0.0057 0.0310 0.0345 -0.0314 -0.0005 0.0042 0.0504 0.0114 -0.0006 0.0078 -0.0128 -0.0097 -0.0053

Clearly, for settings of $p = 250$ causal SNPs, effect sizes are even smaller. The above shows that the simulation settings consist of many small effect sizes.
