# Supplementary material for: Heritability Estimation using a Regularized Regression Approach (HERRA): Applicable to continuous, dichotomous or age-at-onset outcome
Source: PLoS One. 2017 Aug 16;12(8):e0181269. doi: 10.1371/journal.pone.0181269 (PMC5559077; doi:10.1371/journal.pone.0181269)
Supplement: S7 Text — Detailed funding and cknowledgments list. (PDF) [file pone.0181269.s011.pdf]

# Heritability Estimation using a Regularized Regression Approach (HERRA): Applicable to Continuous, Dichotomous or Survival Outcome

Malka Gorfine[1,*], Sonja I Berndt[2], Jenny Chang-Claude[3], Michael Hoffmeister[4], Loic Le Marchand[5], John Potter[6], Martha L Slattery[7], Nir Keret[1], Ulrike Peters[6], Li Hsu[6,*]

**1 Department of Statistics and Operation Research, Tel Aviv University, Tel Aviv, Israel**

**2 Division of Cancer Epidemiology and Genetics, National Cancer Institute, National Institutes of Health**

**3 Division of Cancer Epidemiology, German Cancer Research Center, Heidelberg, Germany**

**4 Division of Clinical Epidemiology and Aging Research, German Cancer Research Center, Heidelberg, Germany**

**5 Epidemiology Program, University of Hawaii Cancer Center**

**6 Public Health Sciences Division, Fred Hutchinson Cancer Research Center, Seattle, WA**

**7 Department of Internal Medicine, University of Utah Health Sciences Center**

*** Correspondence: gorfinem@post.tau.ac.il, lih@fredhutch.org**

# S11 Text: Funding and acknowledgments

R01 CA189532, Drs Li Hsu and Malka Gorfine.

COLO2&3: National Institutes of Health (R01 CA60987, Dr. Loic LeMarchand).

DACHS: German Research Council (Deutsche Forschungsgemeinschaft, BR 1704/6-1, BR 1704/6-3, BR 1704/6-4 and CH 117/1-1), and the German Federal Ministry of Education and Research (01KH0404 and 01ER0814).

DALS: National Institutes of Health (R01 CA48998 to Dr Martha L Slattery).

MEC: National Institutes of Health (R37 CA54281, Laurence N Kolonel; P01 CA033619, and R01 CA63464).

PLCO: Intramural Research Program of the Division of Cancer Epidemiology and Genetics and supported by contracts from the Division of Cancer Prevention, National Cancer Institute, NIH, DHHS. Additionally, a subset of control samples were genotyped as part of the Cancer Genetic Markers of Susceptibility (CGEMS) Prostate Cancer GWAS (Yeager, M et al. Nat Genet 2007 May;39(5):645-9), Colon CGEMS pancreatic cancer scan (PanScan) (Amundadottir, L et al. Nat Genet. 2009 Sep;41(9):986-90 and Petersen, GM et al Nat Genet. 2010 Mar;42(3):224-8), and the Lung Cancer and Smoking study. The prostate and PanScan study datasets were accessed with appropriate approval through the dbGaP online resource (http://cgems.cancer.gov/data/) accession numbers phs000207v.1p1 and phs000206.v3.p2, respectively, and the lung datasets were accessed from the dbGaP website (http://www.ncbi.nlm.nih.gov/gap) through accession number phs000093 v2.p2. Funding for the Lung Cancer and Smoking study was provided by National Institutes of Health (NIH), Genes, Environment and Health Initiative (GEI) Z01 CP 010200, NIH U01 HG004446, and NIH GEI U01 HG 004438. For the lung study, the GENEVA Coordinating Center provided assistance with genotype cleaning and general study coordination, and the Johns Hopkins University Center for Inherited Disease Research conducted genotyping.

VITAL: National Institutes of Health (K05 CA154337).

WHI: The WHI program is funded by the National Heart, Lung, and Blood Institute, National Institutes of Health, U.S. Department of Health and Human Services through contracts HHSN268201100046C, HHSN268201100001C, HHSN268201100002C,

HHSN268201100003C, HHSN268201100004C, and HHSN271201100004C.

GECCO: National Cancer Institute-U01 CA137088, U01 CA164930, U01 CA185094.

GECCO: The authors would like to thank all those at the GECCO Coordinating Center for helping bring together the data and people that made this project possible.

DACHS: We thank all participants and cooperating clinicians, and Ute Handte-Daub, Renate Hettler-Jensen, Utz Benscheid, Muhabbet Celik and Ursula Eilber for excellent technical assistance.

PLCO: The authors thank Drs. Christine Berg and Philip Prorok, Division of Cancer Prevention, National Cancer Institute, the Screening Center investigators and staff or the Prostate, Lung, Colorectal, and Ovarian (PLCO) Cancer Screening Trial, Mr. Tom Riley and staff, Information Management Services, Inc., Ms. Barbara O'Brien and staff, Westat, Inc., and Drs. Bill Kopp, Wen Shao, and staff, SAIC-Frederick. Most importantly, we acknowledge the study participants for their contributions to making this study possible.

WHI: The authors thank the WHI investigators and staff for their dedication, and the study participants for making the program possible. A full listing of WHI investigators can be found at:
https://cleo.whi.org/researchers/Documents%20%20Write%20a%20Paper/WHI%20Investigator%20Short%20List.pdf
